# A southern African archaeological database of organic containers and materials, 800 cal BC to cal AD 1500: Possible implications for the transition from foraging to livestock-keeping

**DOI:** 10.1371/journal.pone.0235226

**Published:** 2020-07-08

**Authors:** Faye Lander, Thembi Russell

**Affiliations:** School of Geography, Archaeology and Environmental Studies, University of the Witwatersrand, Johannesburg, South Africa; Universita degli Studi di Milano, ITALY

## Abstract

Although evidence of organic materials has consistently been reported in the archaeology of southern Africa little attention has been given to how this evidence, so slight in comparison to pottery and lithics, might be used to understand the transition from foraging to livestock-keeping in southern African Archaeology. We have compiled a geo-referenced, radiocarbon database of these organic, material culture remains, with particular reference to containers made of ostrich eggshell, wood, gourd, tortoise shell, twine, and leather over a 2300-year period to capture the periods before and after the appearance of livestock. We have mapped the organic materials for the period 800 cal BC to cal AD 1500 and explored the subsistence base of those who used them. This distribution is compared to that of pottery and livestock remains–conventionally the two archaeological markers of pastoralists. The paper interrogates what this might add to the vexed question of how the practice of livestock-keeping and pottery-making spread into and through the region (the hunter-herder debate). Our analysis suggests that ostrich eggshell containers can be used as a proxy for hunter-gatherers. By comparing areas of bead *manufacture* with those that have evidence only of bead *use*, we show the areas to which items may have travelled, along already established hunter-gatherer exchange networks. Our results suggest that hunter-gatherers widely and quickly adopted pottery across southern Africa in a process of cultural diffusion and local innovation, and that this was possibly the main mechanism for the dispersal of livestock at 2100 years ago.

## Introduction

One of the key transitions in human history is from foraging to food production. In southern Africa, the subsistence base of the autochthonous people was one of hunting and gathering until approximately 2100–2300 years ago. The first appearance of domestic plants, animals and pottery is linked to two spread events. The first was of livestock without cultivation to the drier western half of southern Africa. Sites with livestock have proved to be difficult to distinguish from those without livestock because both have a similar archaeological “Later Stone Age” signature of stone tools, some pottery, a large wild faunal assemblage, low numbers of livestock and ostrich eggshell beads. The second, slightly later and less controversial spread is that of agro-pastoralist, metal-working (‘Iron Age’) Bantu-language speaking farmers into the eastern summer rainfall area. The higher archaeological visibility of these farmer sites (highly decorated thick-walled pottery, hut floors, and iron working) makes it easier to distinguish them from those of hunter-gatherers.

In southern Africa, the ‘hunter-herder’ debate is about whether the spread of novel traits was associated with the migration of people (demic diffusion) [1–15] or by local innovation or their adoption by hunters and gatherers (cultural diffusion) [16–31]. The paper is situated within this debate.

We use the contrasting categories ‘cultural/demic’ and ‘hunter/herder’ as useful heuristic devices [32]. It is widely appreciated that these are not strictly bounded categories. Herder is used to describe the practice of keeping livestock (whilst retaining a hunter-gatherer worldview and subsistence base [31, 33–35]–we prefer the term livestock-keeper for this category). Hunter-gatherer, farmer and pastoralist are used to describe the worldview that accompanies hunting and gathering, sedentary agro-pastoralism and livestock-ownership respectively [36, 37]. The prevailing consensus is that the spread of novel traits throughout southern Africa was by immigration, imitation and innovation [36, 37, 38]. How to distinguish these in the archaeological record remains unclear [39–41].

**Fig 1:**
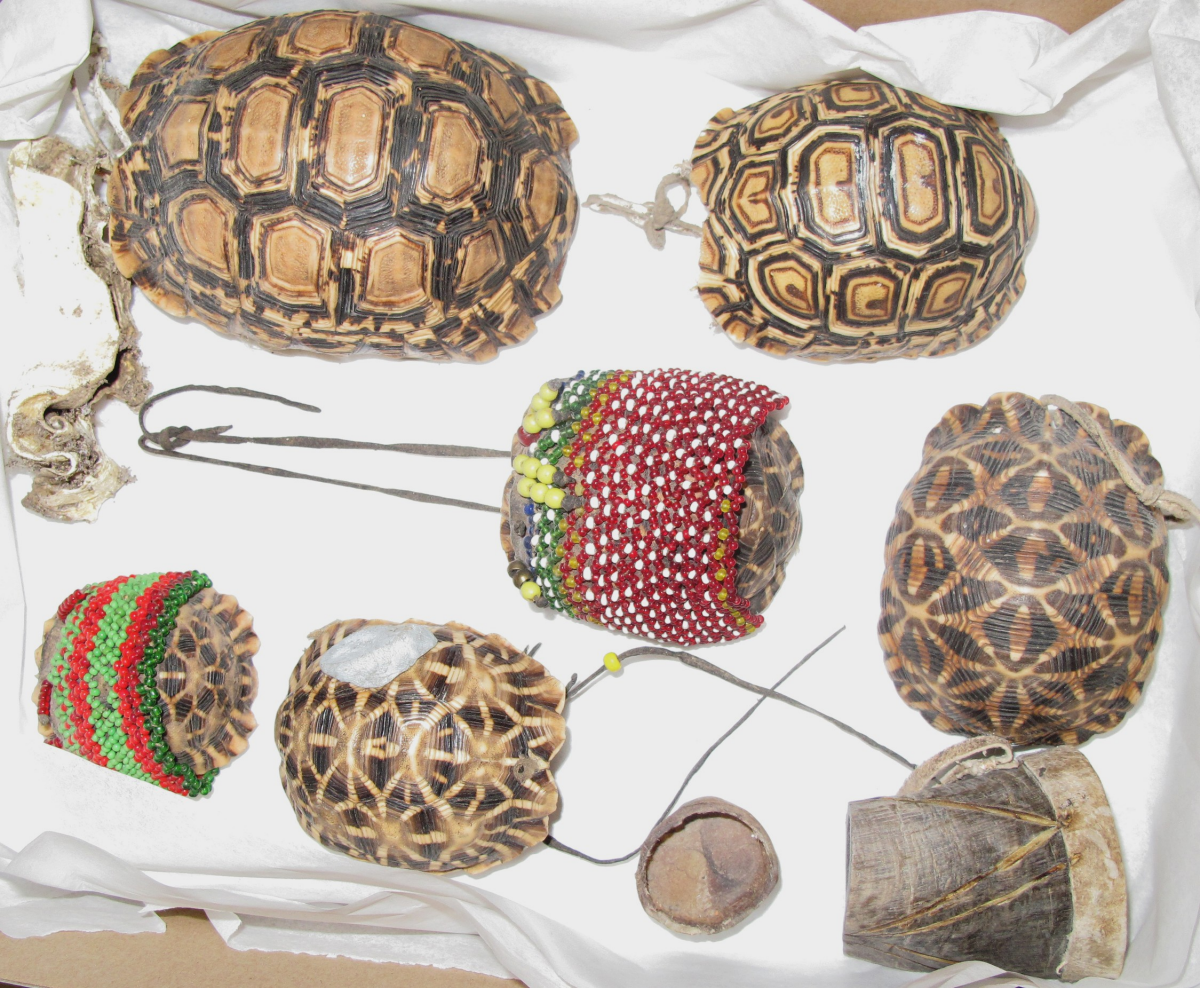
Examples of ethnographically recorded organic containers: Six containers made from tortoise shell and one from bone, Central Kalahari from Ghanzi farm Bushmen, Botswana 1973 (Unaccessioned, Russell and Russell Kalahari Collection, University of the Witwatersrand, South Africa) [42].

**Fig 2:**
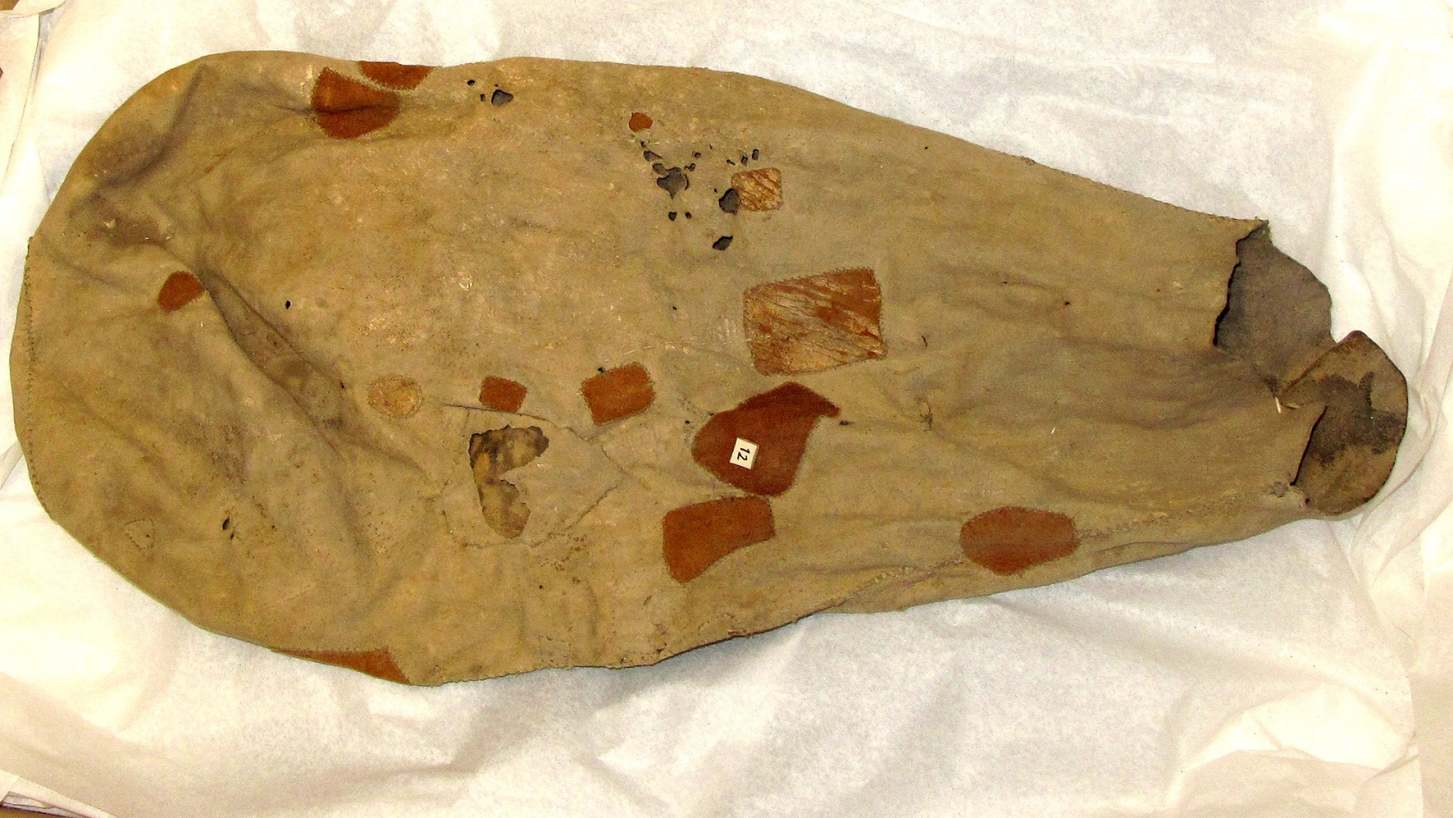
Examples of ethnographically recorded organic containers: A leather bag made by Ghanzi farm Bushmen, Botswana, 1973 (Unaccessioned, Russell and Russell Kalahari Collection, University of the Witwatersrand, South Africa) [42].

**Fig 3:**
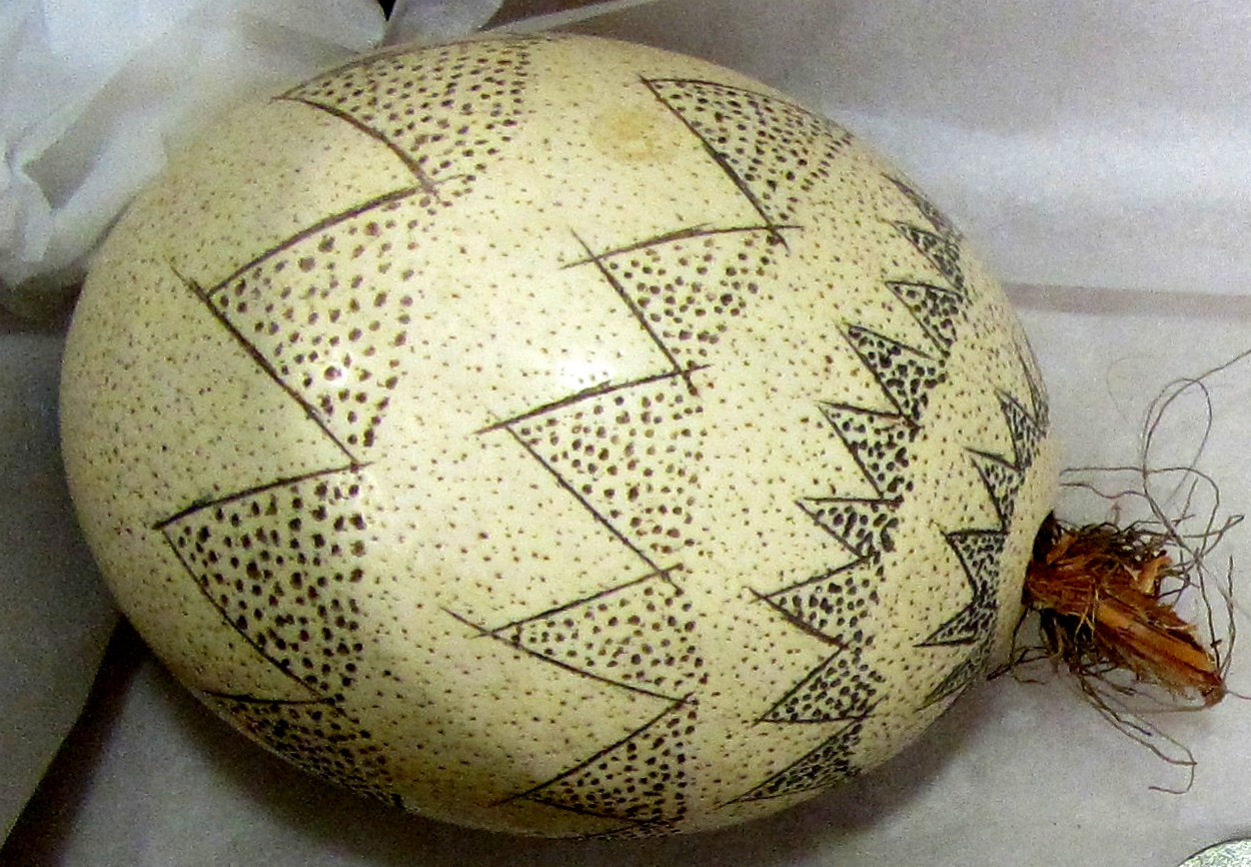
Examples of ethnographically recorded organic containers: A decorated ostrich eggshell made by Ghanzi farm Bushmen, Botswana, 1973 (Unaccessioned, Russell and Russell Kalahari Collection, University of the Witwatersrand, South Africa) [42].

We start from the premise that the spatio-temporal distribution of organic containers might carry information on the mode of subsistence of their users. Although the southern African ethnographic and historic record is rich in accounts of the use of containers made from a variety of organic raw material by farmers, pastoralists and hunter-gatherers, the archaeological evidence of such use has not previously been considered in the debate (Figs 1–3).

Our interest was piqued by Sadr and Sampson’s [43] suggestion of a similarity between the earliest thin-walled pottery as found at a number of sites in southern Africa from approximately 2300 years ago [43–46] and ostrich eggshell containers: “Indeed, given some of the vessel forms in the earliest ceramic levels of Die Kelders (Schweitzer 1979: figs. 25 and 28), we wonder whether the thin-walled pots were not initially meant to imitate ostrich eggshell containers” [43:246]. Although not explicit, their interpretation favours potting as a local innovation amongst hunter-gatherers, since ostrich eggshell containers are conventionally interpreted as a hunter-gatherer item [47–50]. We were curious as to what the motivation for such imitation might be, and whether imitation of function necessarily follows form [cf. 51:192]. We subsequently looked for evidence of the use of ostrich eggshell containers by both southern African pastoralists and farmers and of the co-occurrence in time and space of ostrich eggshell containers and early pottery.

We assembled and mapped geo-referenced archaeological data relating to organic container manufacture and/or use for the period 800 cal BC to cal AD 1500 alongside archaeological data on the occurrence of livestock and pottery (without cultivation). We also included ostrich eggshell beads.

The presence in southern Africa of prehistoric exchange networks amongst some hunter-gatherer groups has been established [52–55]. Ostrich eggshell beads, for example, may have been exchanged over some distance [52–55]. In an effort to understand what the archaeological distributions of material items might mean, we also captured the spatio-temporal data for the occurrence of ostrich eggshell beads in order to compare it to that of ostrich eggshell containers. We expected ostrich eggshell containers, an item that is not commonly exchanged, to have a restricted distribution whereas beads, the most exchanged item, as recorded ethnographically, would travel more widely [cf. 53, 56, 57]. Where possible we compared the availability of particular raw materials with the archaeological distribution of an associated item.

In compiling the databases we were driven by the following questions:

1. What is the spatio-temporal distribution of organic containers in southern African archaeology for this period and what are its implications? Which type of container is the oldest?
2. Are particular types of container associated with particular modes of subsistence (typically characterised as hunter-gatherers, farmers or pastoralists)?
3. How does the distribution of organic containers compare to that of thin-walled pottery and of livestock?
4. Do ostrich eggshell beads and ostrich eggshell containers have the same spatio-temporal distribution?
5. Is there an environmental limitation to the distribution of ostrich eggshell and gourd (*Lagenaria sp*.) containers?

## Materials

The paper presents two geo-referenced databases. The first database captures organic, archaeological material recovered from Later Stone Age sites that may have been used in container manufacture, namely, ostrich eggshell, tortoise shell, wood, twine, gourd and leather (Database 1, S1 Appendix). In addition, data on the occurrence of ostrich eggshell beads, pottery and livestock, from non-agriculturist contexts only, have been included. It excludes all data from agro-pastoralist (farmer) sites (associated with the first use of iron and speakers of Bantu-languages), with the exception of pottery related to farmers at Later Stone Age sites. The second database presents written accounts of the use of organic containers among Khoesan and Bantu-language speaking groups as recorded since the 16^th^ century (S1 Appendix).

### Database 1: Southern African archaeological organic database, 800 cal BC- cal AD 1500

Database 1 provides an inventory of the six types of organic remains recovered from the southern African Later Stone Age context from cal 891 BC to cal AD1512 (for ease of reference and because of the lack of precision in radiocarbon dates, we refer to this as 800 cal BC to cal AD 1500 in the headings) (S1 Map). The geographic scope includes regions between latitudes 17 to 34 degrees south and between longitudes 11 to 33 degrees east. This includes coverage of Namibia, Botswana, Zimbabwe, South Africa, Lesotho and the Kingdom of Eswatini (formerly Swaziland). We excluded Zambia because of the limited reporting/research and/or the absence of organic remains from sites in Zambia for the period in which we are interested. We could find no evidence of ostrich eggshell containers although there are sites with ostrich eggshell beads [58–62].

For purposes of discussion within the hunter-herder debate, the database also contains radiocarbon-dated and geo-referenced data from all known occurrences of Later Stone Age pottery, livestock and ostrich eggshell beads for the same period.

### Database 2: Southern African historical database of organic container use, 16^th^ century onwards

Database 2 presents the spatio-temporal distribution of organic container use amongst extant and historical Khoesan populations, as well as Bantu-language speaking agro-pastoralists, as derived from ethnographic and historic accounts ranging in date from the 16^th^ century AD to the present.

## Methods

The maps were made using ArcGIS version 10.5. The base map was built using the freely available 4.01 Biomes in Africa Map and a more detailed vector geospatial vegetation/biome dataset for South Africa, Lesotho and Swaziland from the South African National Biodiversity Institute.

### Compilation of the southern African archaeological organic database, 800 cal BC- cal AD 1500 (Database 1)

This database was collated from multiple sources, namely, site excavation reports, academic publications, unpublished dissertations and radiocarbon laboratory lists (S1 Table). Key texts were [63] for Later Stone Age sites, and [64] for sites related to the spread of livestock keeping. The database contains the following fields:

1. A unique site reference number.
2. The official site name as designated by the excavator(s).
3. The type of site (open or shelter, coastal or inland).
4. Country and/or province location.
5. Geographic co-ordinates.
6. The subsistence base of the site’s occupants.
7. Stratigraphic context of radiocarbon date and/or material remains.
8. Uncalibrated radiocarbon date and laboratory number. Direct dates are highlighted in bold alongside the material dated.
9. Calibrated date ranges. Dates were calibrated using Oxcal version 4.3. All materials were calibrated using the Southern Hemisphere Calibration Curve ShCal 13 [65]. Dates derived from uncorrected and uncalibrated marine shell are calibrated using Marine13 [66]. The mean marine carbon reservoir correction (∆R) is taken [67], with a weighted mean of 146±85 for the western coast of southern Africa (this applies only to the coastal regions of Namaqualand in the Northern Cape and the Western Cape Province). For the southern coast of South Africa, including the coastal regions between Mossel Bay and Port Elizabeth, the mean marine carbon reservoir correction (∆R) is taken, with weighted mean ∆R of 187±18 [68]. All calibrations are to one sigma and reported as calibrated years cal BC/cal AD. A reservoir correction is not used for dates derived from ostrich eggshell, since they are fossilised and thus old (Higham pers comm. 2019).
10. Radiocarbon dates were given a rating:
  1. Good. A direct date.
  2. Fair. Sequence of dates with good stratigraphic control and the excavator is confident in the association between dates and material remain(s).
  3. Poor. Date derives from poor stratigraphic contexts or there is only a single date obtained for a site.

The sites are grouped temporally into four 600-year time slices:

1. Group 1: 891–291 cal BC.
2. Group 2: 290 cal BC- cal AD 310.
3. Group 3: cal AD 311–911.
4. Group 4: cal AD 912–1512.

These four 600-year time slices capture patterns that have been identified previously [25, 28, 38, 42, 64, 69]–an early first millennium AD appearance of livestock and ceramics (Groups 2 and 3) and a later first millennium to second millennium AD appearance of lugged ware, livestock and large ostrich eggshell bead diameters associated with Khoe pastoralists (Group 4). Each map has an accompanying table (arranged as separate sheets in S1 Appendix) showing the finer chronology of the material culture for each time slice.

The reporting style of published excavated material assemblages is uneven. Gaps occur particularly in the reporting of the quantity, size and presence or absence of the manufacture of ostrich eggshell beads. Where individual counts of material items could not be established for each stratigraphic level, total counts at a site are reported. The database was filtered to remove sites with no clear association between stratigraphic context and dates, or which fell outside of the geographic scope of the database collection–e.g. northern Mozambique where sites lie above 17 degree latitude. A total of 49 sites and 98 radiocarbon dates were thus excluded from the database (S1 Appendix). Post-filtering, the database comprises 187 sites, associated with 489 radiocarbon dates. Of these 77 sites contain organic remains (S1 Appendix).

For ease of reference and because there are so few organic remains, the finds in Database 1 are condensed into five categories:

1. Category 1: Later Stone Age pottery (type of temper).
2. Category 2: Ostrich eggshell containers (mouth/rim fragments and/or decorated pieces).
3. Category 3: All other organic remains, namely leather, twine, gourd (*Lagenaria sp*.), wood or tortoise carapace.
4. Category 4: Livestock.
5. Category 5: Ostrich eggshell beads–quantity, and presence or absence of manufacture (this was based on the presence of unfinished beads).

**Fig 4:**
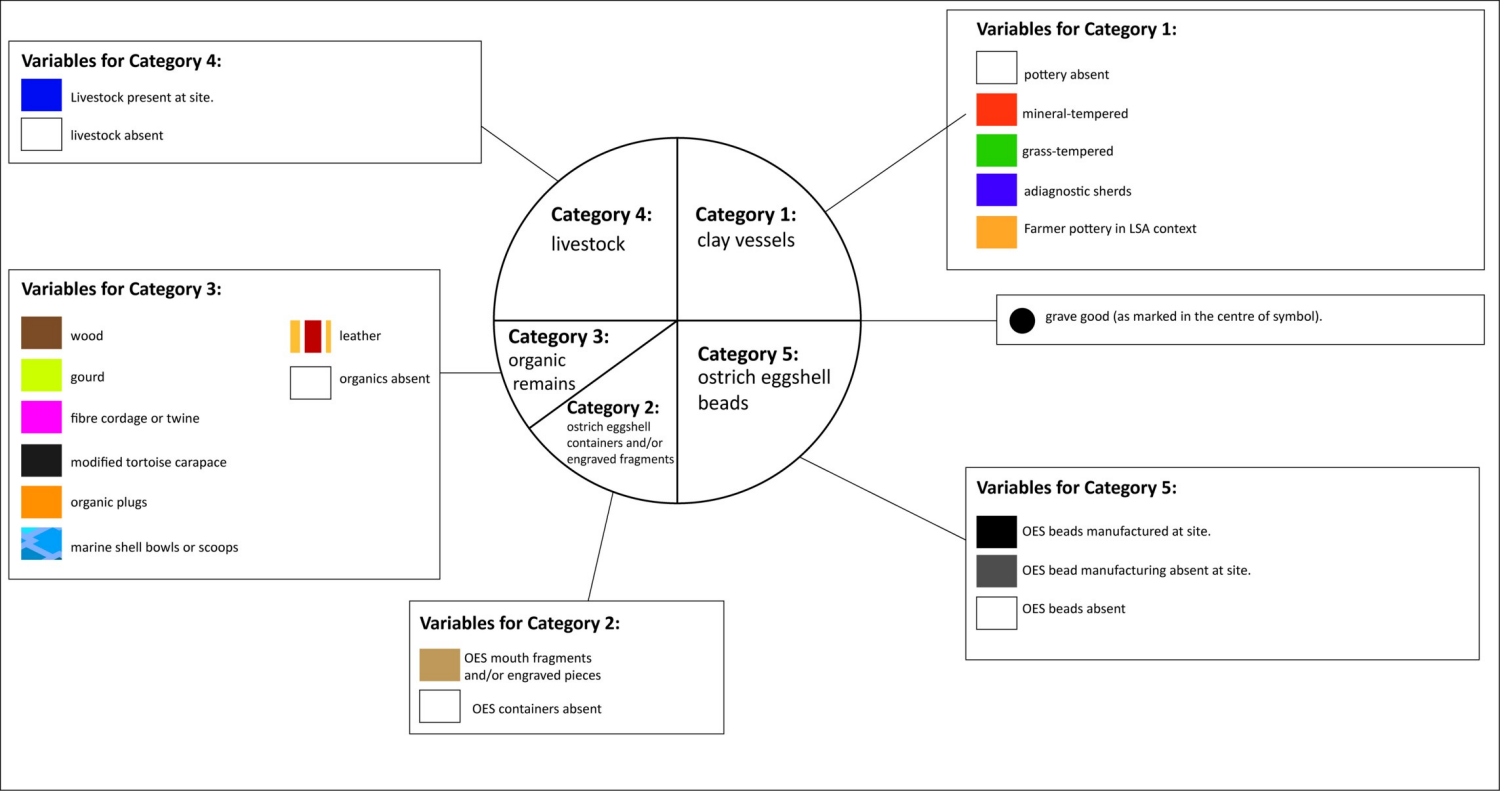
The symbol used to display the five categories of archaeological material found at a site.

On the maps, these categories are reflected in a symbol (Fig 4). The size of the symbol reflects the number of ostrich eggshell beads recovered at a site. Ostrich eggshell was captured as a container only if it showed either, (a) evidence of modification in the form of a mouth or rim piece, or (b) an engraved or painted pattern. The capturing of tortoise carapace was based on whether or not it was described as ‘modified’ by the excavator. The quantities of other items of material culture at each site are recorded in Database 1. Those sites with a poor temporal rating (3) have a symbol with a 60% transparency setting on the maps for easy identification.

For simple comparison with containers made from organic materials, we took the decision to make a single category of Later Stone Age pottery (category 1) and to look at one of its variables (temper) and to simply consider its presence or absence at a site. All the pottery in this category is thin-walled (5–8 mm) and found exclusively at sites of herders, pastoralists and hunter-gatherers [69]. There are regional subsets to this group that differ in terms of their shape and decoration (For example, in the southwestern Cape [21, 28, 70, 71], the South African interior [42, 72, 73]; south-eastern southern Africa [43, 45, 74]; northern Cape, South Africa [75], Eastern Cape, South Africa [76], Namibia [18], Free-State, South Africa [77], Zimbabwe and Botswana [3, 78–80]. These subsets are not considered here.

To document the types of engraving observed on ostrich eggshell fragments and to confirm the presence of ostrich eggshell rim pieces and other organic material from the excavated record, a sample of six sites were selected for further documentation by visiting the museum collections in South Africa (S2 Table). The six sites were chosen because they have evidence of organic containers and pottery (mineral and grass tempered) (S2 Appendix, S3 Appendix, S4 Appendix).

### Compilation of the southern African historical database of organic container use, 16^th^ century onwards (Database 2)

This *non-exhaustive* geo-referenced database comprises 89 entries from historical and ethnographic sources for the use of organic containers amongst non-agro-pastoralist and agro-pastoralist groups in southern Africa (S1 Appendix, S3 Table). References to the use of non-agro-pastoralist mineral- and grass-tempered pottery were also recorded. The database has a date range from 1593 AD to the present (S1 Appendix).

Database 2 contains the following fields:

1. A unique site reference number.
2. Name of record taker.
3. Documentation type (e.g. traveller account, museum object or photographic collection etc.).
4. Date of record.
5. Cultural group.
6. Mode of subsistence.
7. Region and country of record.
8. The area from which geographic co-ordinates were taken for mapping. Geographic co-ordinates are estimates: none of the accounts provided latitude and longitude information.
9. Geographic co-ordinates.
10. Container type recorded.
11. Container function.
12. Other information (e.g. plant species, where provided).
13. Verbatim record.
14. Reference/source.

### Ethnographic sources

Six ethnographic works were consulted. Five of these are records made among Khoe- and non-Khoe-speaking Bushmen groups between 1950 and 1993 [56, 81–84]. They cover a geographic region that extends from the Nyae Nyae Conservancy in north-eastern Namibia to Ghanzi, in Botswana. The sixth source is Hoernlé’s ethnographic study of the Nama of Walvis Bay, Namibia (1912 and 1923) [85].

### Historical sources

Two online museum archives were accessed: 1. The Oswin Köhler Archive from the Goethe University, Frankfurt (https://www.uni-frankfurt.de/62970506/Collection_Oswin_Köhler). A simple search was carried out by object type or cultural group name between 1911 and 1996. 2. The Pitt Rivers Museum collection (https://www.prm.ox.ac.uk/collections-online). Search parameters included the photographic collections, Africa continent, southern Africa and ‘Khoisan’ cultural group.

The following research publications were consulted: (1). Bollong et al. [86] provides a review of early colonial traveller and settler accounts for the use of pottery amongst historically observed Khoe pastoralist groups and Bushmen occupying the drier interior and south-western coastal regions of South Africa. (2). Rudner [87] offers additional historical references for the use of pottery amongst non-ago-pastoralist groups. (3). Sydow [88] references the use of organic containers by historically documented Khoe pastoralists, (4). Guenther [84] describes container use amongst the Namib Bushmen, Namibia. (5). Shaw and van Warmelo [89] record the use of baskets, gourds, calabashes and wooden containers amongst Bantu language-speaking agro-pastoralists in the Eastern Cape, South Africa. (6). Hooper [90] records containers used by the Zulu and Swazi of KwaZulu-Natal, South Africa and Eswatini (formerly Swaziland) respectively.

The geographic coordinates in Database 2 are approximate, based on maps where provided, and the geographic region as described in each source. A map was compiled by overlaying point data (each symbol refers to a historic or ethnographic account) onto the freely available UNEP Africa average annual precipitation (mm) map (UNEP, 2002 http://geodata.grid.unep.ch).

## Results

**Fig 5:**
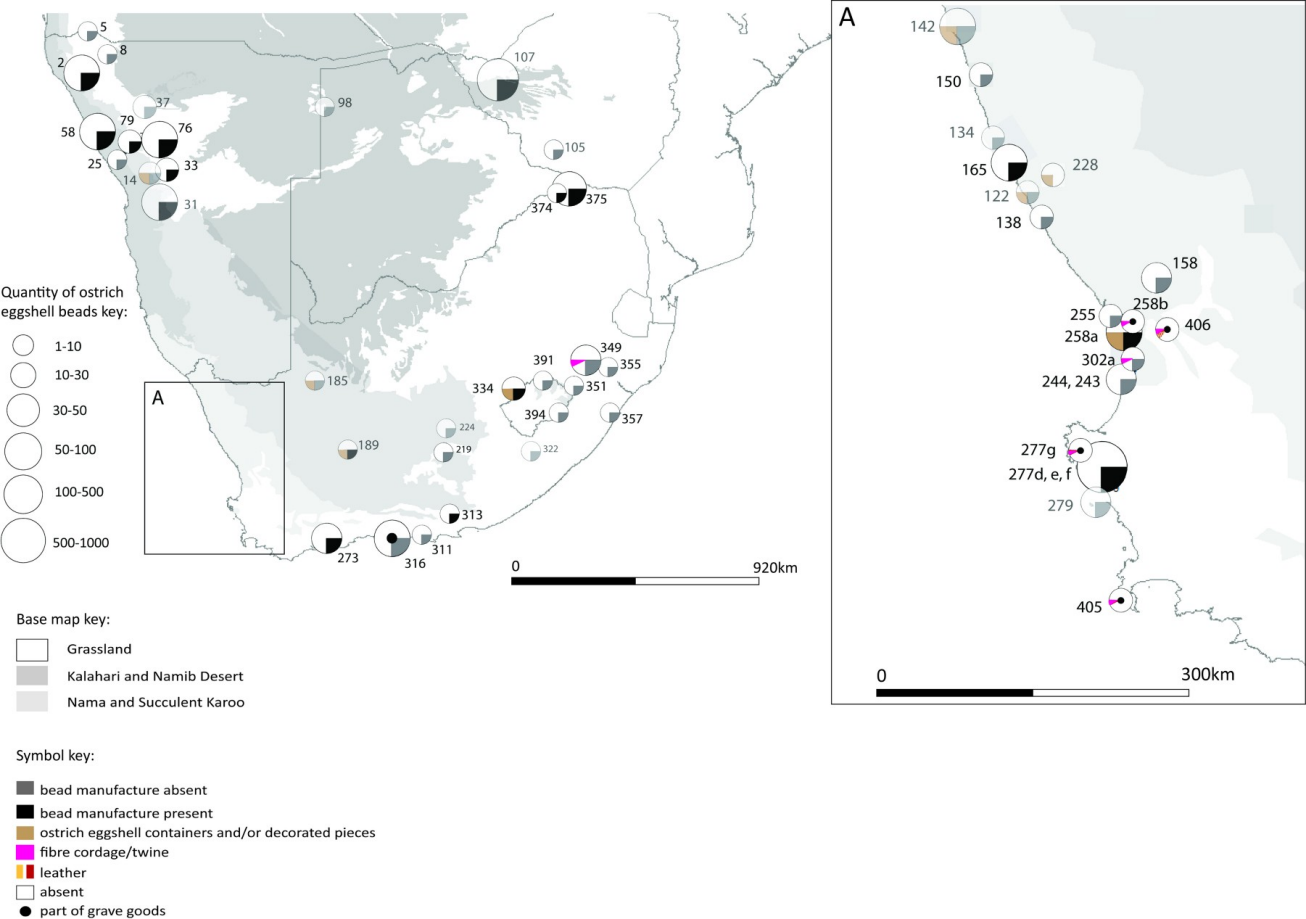
Map 1 showing the distribution of organic finds in the period 891–291 cal BC.

**Fig 6:**
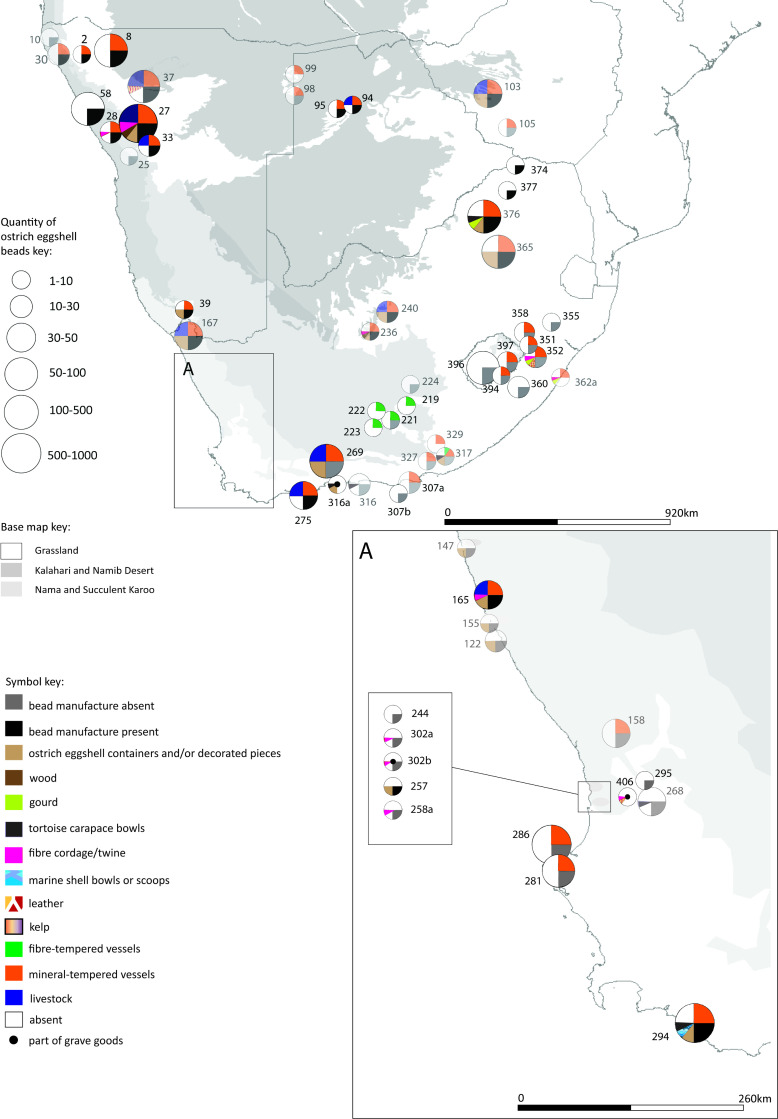
Map 2 showing the distribution of organic finds, livestock and pottery in the period 290 cal BC- cal AD 310.

**Fig 7:**
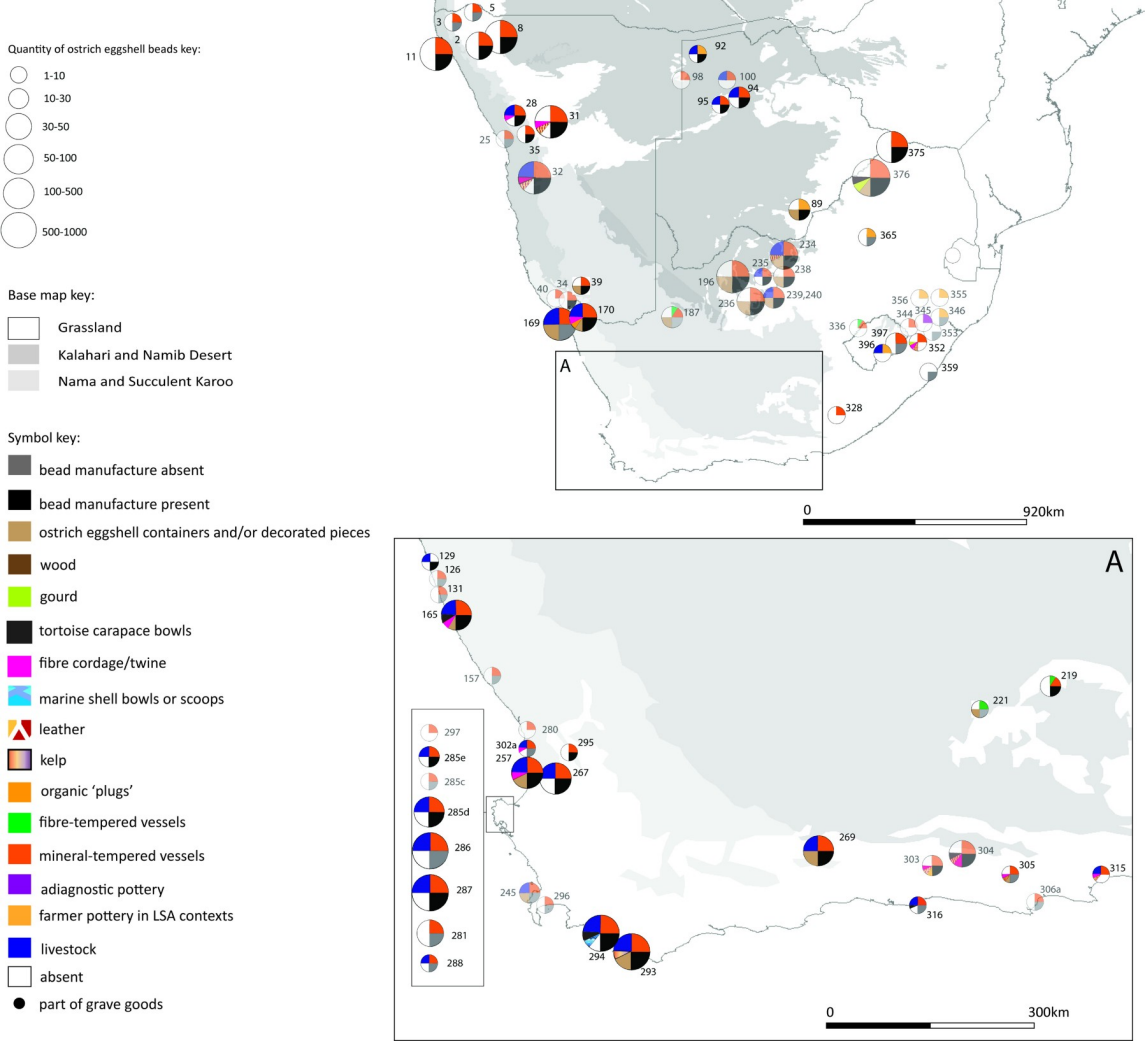
Map 3 showing the distribution of organic finds, livestock and pottery in the period cal AD 311–911.

**Fig 8:**
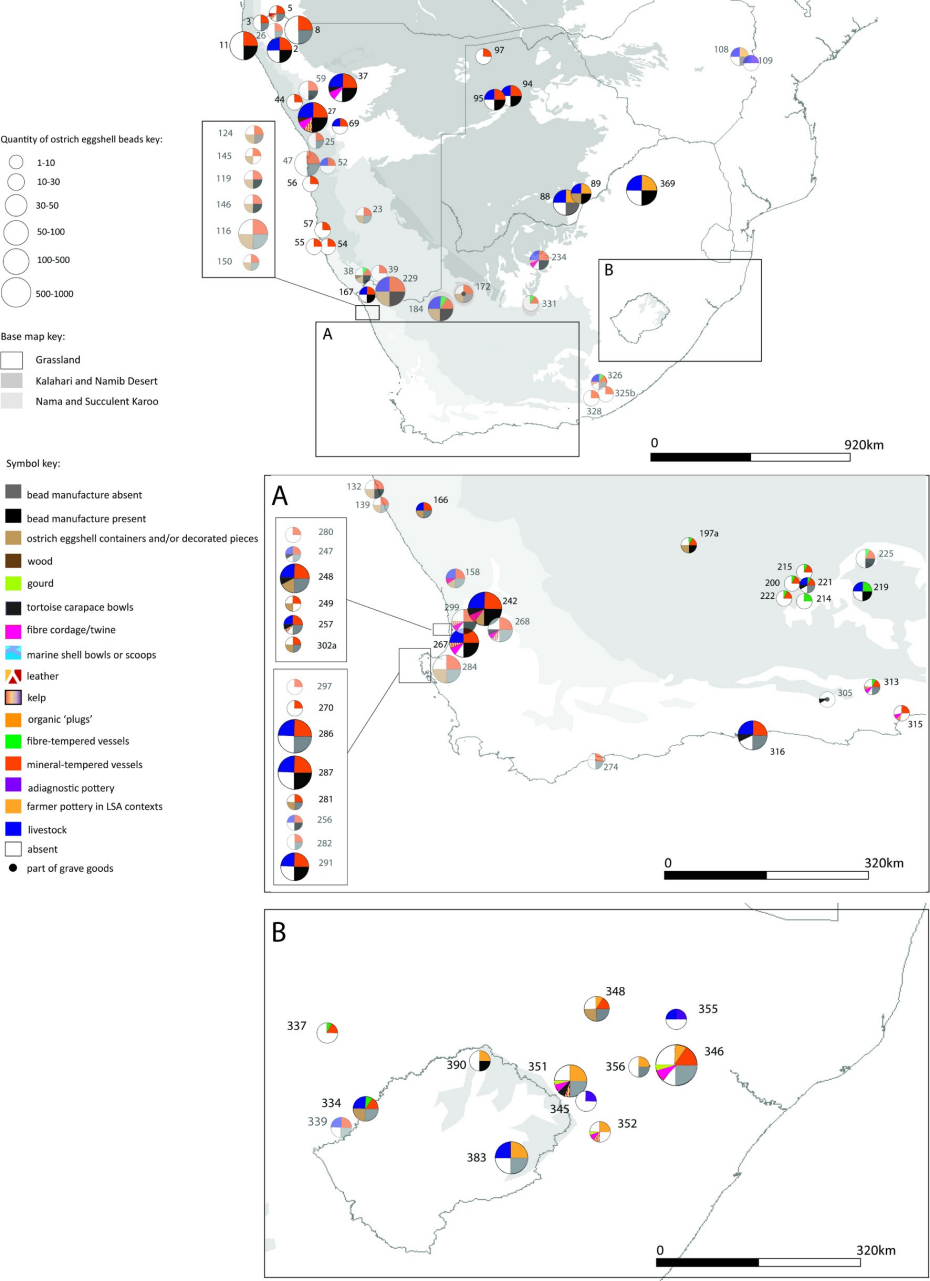
Map 4 showing the distribution of organic finds, livestock and pottery in the period cal AD 912–1512.

Each of the four maps should be read in conjunction with the accompanying spreadsheet (Figs 5–8, S1 Appendix). Key patterns observed for the four time slices are summarised below (Table 1).

**Table 1:** Table showing the occurrence and co-occurrence of archaeological items during each of the four time periods.

Archaeological item	Map 1 (53 sites), 891 -291BC	Map 2 (63 sites) 290 BC- 310 AD	Map 3 (76 sites) AD 311–911	Map 4 (92 sites), AD 912–1512
Ostrich eggshell containers	8	17	18	25
Pottery	0	36 mineral-tempered	61 mineral-tempered	72 mineral-tempered
		5 fibre-tempered	4 fibre-tempered	15 fibre-tempered
Livestock	0	10	28	35
Livestock + bead manufacture	0	9	19	15
Livestock + mineral-tempered pottery	0	10	25	28
Livestock + all types of pottery	0	10	27	35
Sites with beads	48	55	61	64
Sites without beads	5	8	13	26
Mineral-tempered pottery + ostrich eggshell containers	0	12	15	22
Fibre-tempered pottery + ostrich eggshell containers	0	0	2	4
Fibre-tempered pottery + livestock	0	0	0	5
Fibre-tempered pottery + mineral-tempered pottery	0	1	3	13
Ostrich eggshell containers + no pottery	8	5	0	0
Livestock + ostrich eggshell containers	0	6	8	7
No ostrich eggshell containers and no pottery	45	21	3	1
Livestock + no ostrich eggshell beads	0	0	2	4
Livestock + no ostrich eggshell containers	0	4	19	25

### Map 1, 891–291 cal BC

Fifty-three (53) archaeological sites fall within this period (Fig 5). The organic materials present are ostrich eggshell (beads at 48 sites, containers at 8 sites), fibre/twine (6 sites of which 4 are graves) and leather (1 grave site). Five sites contain no evidence of ostrich eggshell beads, four of which are gravesites (this concurs with the observation amongst hunter-gatherers of the Kalahari that all possessions with the exception of beadwork are taken to a person’s grave on death [82:41]) and one is a scatter site. There is no evidence for pottery or livestock during this time slice.

### Map 2, 290 cal BC- cal AD 310

Sixty-three (63) sites fall within this time slice (Fig 6). The organic materials recorded are ostrich eggshell (55 sites with beads, 17 sites with containers), fibre/twine (10 sites, of which 2 are graves), leather (3 sites, of which 1 is a grave), tortoise containers (6 sites), gourd (3 sites), wood (2 sites), and modified marine shell (1 site). Eight sites have no evidence of ostrich eggshell beads. 36 sites contain mineral-tempered pottery; of these sites, 12 also contain ostrich eggshell containers. Five sites contain fibre-tempered pottery–but with no evidence of ostrich eggshell containers, and with very little evidence of ostrich eggshell beads (2 of the sites have less than ten beads each). No fibre-tempered pottery is found with livestock. Five sites have ostrich eggshell containers without pottery. One of these is a grave, three are coastal middens and one is a small coastal rock shelter. All ten sites containing livestock have pottery and beads (all but one of them are bead manufacture sites). Six of the sites with livestock have ostrich eggshell containers (of which four are decorated; one may have been used to store ground ochre) (site 167, Ai Tomas [91: 214]). There is no evidence of bead manufacture at sites in the southern eastern parts of South Africa. Gourds are found only on the eastern side of South Africa.

### Map 3, cal AD 311–911

Seventy-six (76) archaeological sites fall within this period (Fig 7). The organic material recorded is ostrich eggshell (beads at 61 sites, containers at 18 sites), fibre/twine (12 sites), leather (8 sites), tortoise carapace (4 sites), gourd (2 sites), organic plugs (2 sites), kelp (1 site) and marine shell (1 site). Thirteen (13) sites have no evidence of ostrich eggshell beads. 61 sites have mineral-tempered pottery, of these 15 sites also contain ostrich eggshell containers. Four sites have fibre-tempered pottery, 2 of these have ostrich eggshell containers. Fibre-tempered pottery is not found with livestock. Seven sites have agro-pastoralist (farmer) pottery in a Later Stone Age context. There are 28 sites with livestock, of these 25 also have pottery and beads (all but four of these are bead manufacture sites). Eight of the sites with livestock have ostrich eggshell containers. There is no evidence of bead manufacture in KwaZulu-Natal. Gourds are found in the eastern half of southern Africa. Fibre- and mineral-tempered ware are found together at sites for the first time (4 sites only, all without livestock).

### Map 4, cal AD 912–1512

Ninety-two (92) sites are recorded in this period (Fig 8). The organic material recorded is ostrich eggshell bead (64 sites), ostrich eggshell containers (25 sites), fibre/twine (13 sites), leather (12 sites), tortoise carapace (10 sites), gourd (3 sites) and wood (2 sites). Twenty-six (26) sites have no evidence of ostrich eggshell beads. Seventy-two (72) sites have mineral-tempered pottery, 22 of which also have ostrich eggshell containers. Fifteen (15) sites have fibre-tempered pottery, 4 of which also have ostrich eggshell containers. For the first time fibre-tempered pottery is found with livestock (4 sites), at all of these sites the fibre-tempered pottery is found with mineral-tempered pottery. Two sites contain fibre-tempered pottery only. Eleven (11) sites have agro-pastoralist (farmer) pottery in a Later Stone Age Context. Thirty-five (35) sites have livestock, of these 33 have pottery as well as beads (17 of these have evidence of bead manufacture). Seven sites with livestock have ostrich eggshell containers. There is no evidence of bead manufacture in KwaZulu-Natal. Gourds are only found in the eastern portion of South Africa.

### Ethnographic and historic observations of container use from AD 1600 onwards

**Fig 9:**
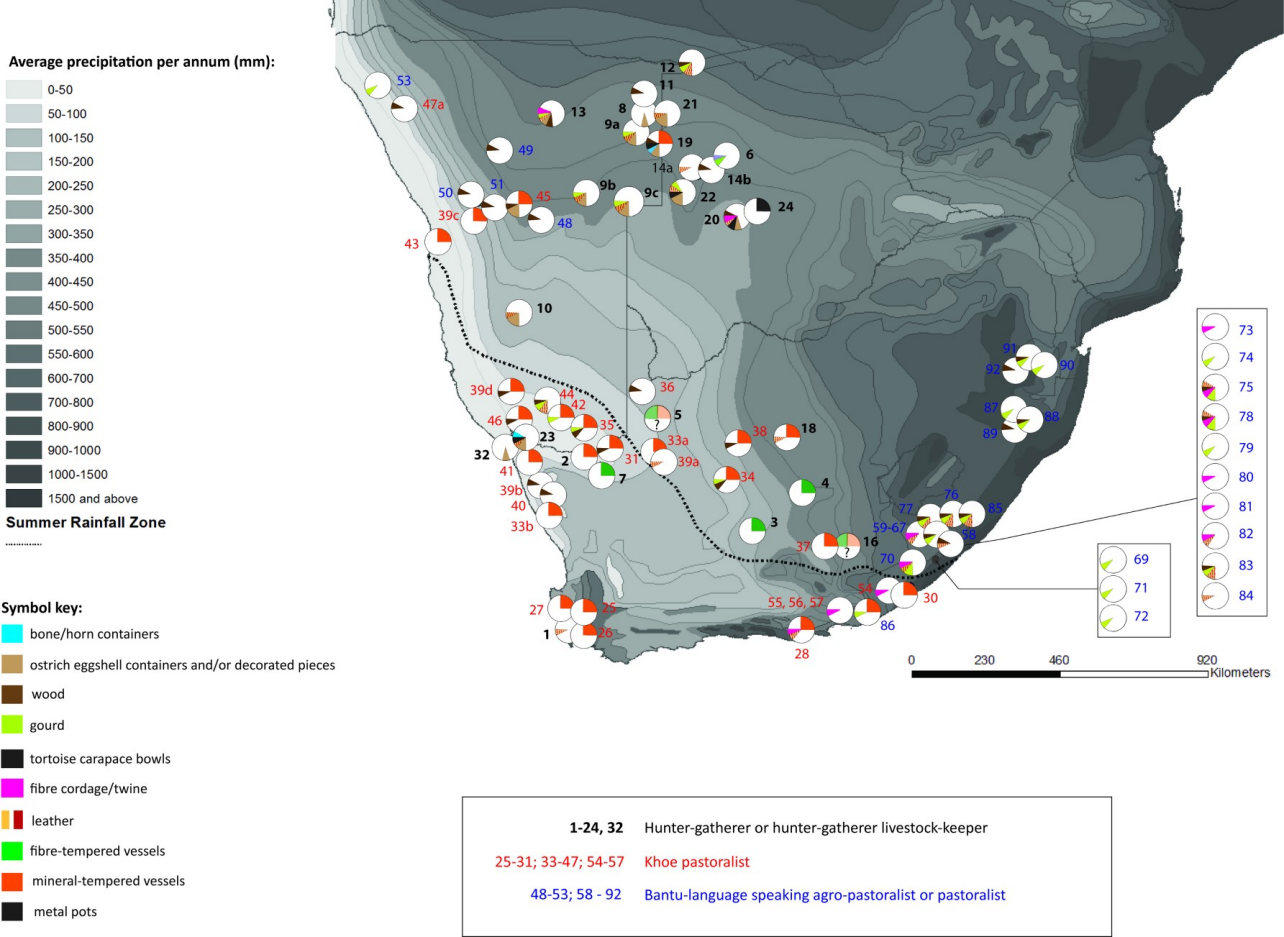
Map to show the ethnographic and historic observations of container use from AD 1600 onwards.

Mistakes are likely to have been made in assigning identity to people encountered on the southern African landscape during the historic period [92]. The map showing the ethnographic/historic use of organic and pottery vessels largely coincides with what is understood from the archaeology: (1) Fibre-tempered pottery was identified only among hunter-gatherers; (2) Both hunter-gatherers and pastoralists used mineral-tempered vessels; (3) Gourds were used by both Bantu-language speakers and some pastoralists. The distribution of gourd use is concentrated in the summer rainfall area of southern Africa; and (4) Ostrich eggshell container use is almost exclusively by hunter-gatherers (Fig 9).

## Discussion

Discussion of leather and plant fibre/twine evidence is limited due to their poor preservation, low numbers, and the impossibility of knowing whether they were used for bags, containers, nets or some other function. The numbers of marine and tortoise containers were likewise low. Our discussion focuses on ostrich eggshell containers which are well-preserved and found in relatively high numbers. We turn first to the questions posed at the start of the paper.

(i) What is the spatio-temporal distribution of organic containers in southern African archaeology for the study period and what are its implications? Which type of container is the oldest?

Organic containers made of ostrich eggshell are found throughout the time-period but with spatial clustering. They are found in their highest numbers along the west coast of South Africa. They are very rare in Namibia, and the southeastern part of South Africa where gourds are found. Evidence of plant fibre and leather is concentrated at gravesites during the period 891–291 cal BC, possibly a consequence of the better preservation in these microenvironments. Later they are found at living sites. Containers made from other materials such as marine and tortoise shell, wood and gourd are found in low numbers from 290 cal BC onwards. Ostrich eggshell containers predate the introduction of pottery. On the available evidence, this is the oldest preserved type of container in southern Africa.

(ii) Are particular types of container associated with particular modes of subsistence (typically characterized as hunter-gatherers, farmers or pastoralists)?

**Fig 10:**
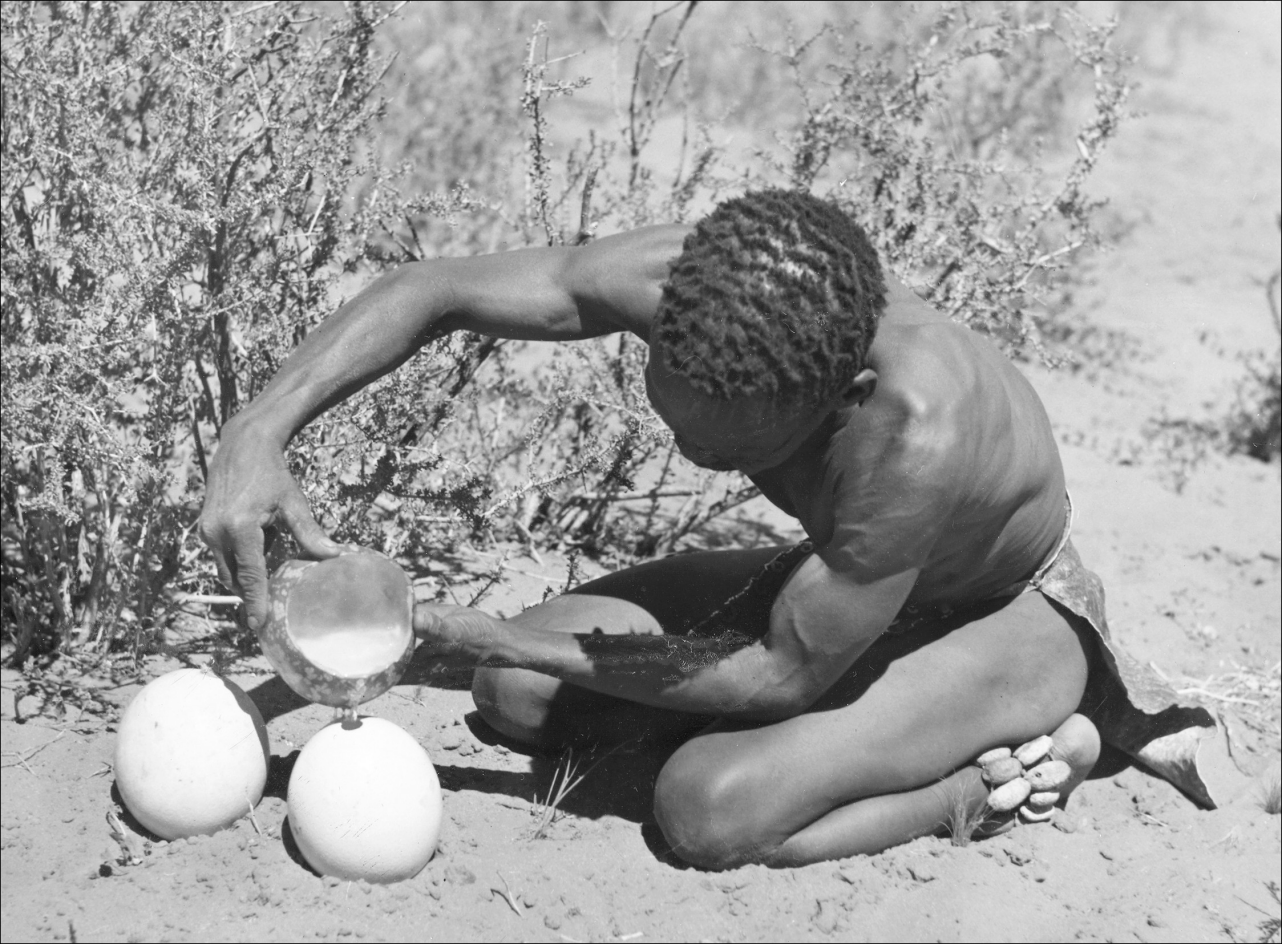
Man filling ostrich eggshells with water for storage, 1945–50, Southern Africa. Copyright Pitt Rivers Museum, University of Oxford (1998.193.33).

Archaeological remains of decorated and undecorated ostrich eggshell containers in hunter-gatherer contexts in southern Africa date back to 60 000 years ago, firmly establishing them as a hunter-gatherer item ahead of the arrival of farming and pastoralism [49, 93–99]. The historic and ethnographic record shows that they were used for carrying and storing water (amongst the G/wi of the central Kalahari, Botswana [82: 221],! Kung of northern Botswana [81: 122]; southern African Bushmen [100: 143],! Kung of Nyae Nyae, Namibia [56: 77], and the Kalahari Bushmen [101: 28]) (see also [85, 86, 88]) (Fig 10). Two historic records by European travellers of the use of ostrich eggshell water containers by hunter-gathers are noted by [88: 24], by Hans Schinz (1884/1887) of the Kalahari Bushmen and by J.H. Wilhelm of the! Kung Bushmen. We came across only one historic record of this use among southern African pastoralists–Vedder’s observation in 1938 of Nama in Namibia with pottery, wooden milk buckets and ostrich eggshells for water containers [88:30]. No ethnographic accounts were found of their use by either southern African pastoralists or agro-pastoralists. Ostrich eggshell beads, however, are common among all three subsistence groups [102]. We discovered only a handful of ethnographic and historic accounts of the use of ostrich eggshells among African farmers or pastoralists outside of southern Africa (on Konso homestead rooftops in Ethiopia [103], as a symbolic household ornament associated with marriage and fertility among Somali and Tuareg pastoralists [50] and as a cooking fat container by the Ingessa (Gaam), Sudan) [104].

We were interested in why, and if, these portable and durable water containers remained an exclusively hunter-gatherer item after the arrival of food production. There is some evidence in the Later Stone Age southern African archaeology that they were used to store materials other than water (ochre [91, 105–107], specularite [93, 108] and ostrich eggshell pieces [109]). Overwhelmingly they are documented as containers for water -“water flasks” [48, 49,100, 110, 111].

They are used on foraging trips, to bring water back to camp and to store water in times of plenty. Buried caches of as many as ‘several hundred’ have been recorded among the G/wi in Botswana for use in the dry season [81] (see also [82, 112]). Marshall [56: 5] notes of the! Kung of Nyae Nyae Namibia, “They took ostrich eggshells filled with water and stayed gathering for two or three days, and returned to /Gam when their shells were empty”. They are items that are personally owned and sometimes engraved to mark ownership [49, 56, 81].

It is in their use as water containers that an argument can be built as to why ostrich eggshells are not a pastoralist or an agro-pastoralist item: they are too small. The capacity of an ostrich eggshell is about 1 litre [56, 81].! Kung families had 8 to 10 eggshell containers each [56]. They are portable and durable, but have the disadvantage of being heavy (about 0.5 kg when empty) [56]. The alternative is to use animal bladders or stomachs which are less popular than ostrich eggshells as they tear easily [81]. They are not suitable as water containers for households with livestock. Young livestock, which must remain at the homestead, require daily watering. Leather water bags of 15 litre capacity are required to meet the needs of people and very young animals at the camps of pastoralists in Niger [113: 1] and amongst the Tuareg of West Africa, where:

“…the exact placement of the camp… is determined by the water requirements of the young animals which remain inside the camps, as well as those of the people themselves. Lambs, kids and humans do not require great quantities of water (sufficient amounts can be carried in leather water bags for two days’ supply) but since calves drink more they must be taken directly to the water source every day” [114: 85].

It has been estimated that the daily waters requirement of cattle is 30 to 60 litres per head per day, and that for sheep and goats is 3 to 15 litres [115]. These figures go up in hot weather [115]. These high demands of livestock for daily watering means that pastoralists must live close to water [85, 113, 114, 116]. When the household requirements cannot be met at the water source, gourds and leather bags are used for carrying water [117]. The use of livestock as draught animals means that larger quantities of water can be carried back to the homestead [113]. Grillo [118] working among the Samburu pastoralists records their use of livestock stomach bags for fetching household water and also the use of larger leather pouches in which several gourds could be carried at once. “Most pastoralist Samburu women would tell me that they never stored water at all, and if they did it would be in wooden containers, gourds, or woven containers…” [118:135]. Amongst Somali pastoralists, the average water container made from plant material has a capacity of 30 to 34 litres and is used for fetching and storage [50].

The evidence suggests that in southern Africa, ostrich eggshell containers were a *hunter-gatherer* item. Unlike beads, they were not a regular part of symbolic exchange networks (cf. [119]).

The spatio-temporal mapping shows that pottery is common from its first appearance in southern Africa, approximately 2300 years ago. Its co-occurrence with ostrich eggshell containers at Later Stone Ages sites shows a widespread uptake from its first introduction (cf. [42, 69]). Once pottery arrived in southern Africa, it rapidly became a ubiquitous item at hunter-gatherer sites suggesting that is offered an immediate adaptive advantage to hunter-gatherers. What that was, is impossible to say without residue analysis of potsherds [120–125]. It seems likely that ostrich eggshells were used for water storage and that pottery was used for boiling and rendering. We can postulate that food would have been mainly roasted but not boiled prior to the introduction of pottery. An alternative way of boiling has been recorded–stone boiling, where heated stones are placed within containers made from woven grass, wood or leather [118]. In the later 18^th^ century eastern Cape, Khoe pastoralists are observed using this method, “They boil the food in water in skin bags with stones heated to glowing point” [88: 18]. Pottery would have improved the diet because fat and nutrients, captured by pottery, are lost when roasting on a fire. Pottery would have also allowed the rendering and storage of fat, which would have prolonged its edibility during the hot summer months [126] (cf. [125, 127, 128]). We do not know whether hunter-gatherers were making pottery, although this is very likely [42, 125].

(iii) How does the distribution of organic containers compare to that of thin-walled pottery, and with livestock?

From 290 cal BC when pottery first appears in the record, most sites with evidence of ostrich eggshell containers also have evidence of pottery (12 sites with co-occurrence, 5 sites with ostrich eggshell containers only). Towards the eastern interior and coastal region of South Africa, where there is no ostrich eggshell container use, pottery is found at most sites, sometimes with evidence of gourds. These patterns persist to AD 1512.

From the earliest appearance of livestock at approximately 2100 years BP, there is the co-occurrence of livestock remains with ostrich eggshell containers (6 of the 10 sites with livestock also have ostrich eggshell containers). Organic finds other than ostrich eggshell are low at livestock bearing sites: leather at 1 site, wood at 1 site, fibre at 2 sites. Sites in Botswana and Namibia with evidence of livestock have evidence of leather and twine but no ostrich eggshell containers. From 912 AD, when livestock sites are at a peak, very few also have evidence of ostrich eggshell containers. Leather and fibre/twine are more common at these livestock sites from 912 AD, but the numbers of organic remains are low.

(iv) Do ostrich eggshell beads and ostrich eggshell containers have the same spatio-temporal distribution? And (v) Is there an environmental limitation on ostrich eggshell and gourd container distribution?

Unlike ostrich eggshell containers, which are found only in the areas with a viable habitat for ostriches, ostrich eggshell beads are found beyond their natural habitat. This confirms the suggestions made previously [53, 129].

While ostrich eggshell beads are found at most Later Stone Age sites, evidence of bead manufacture is rarer. Sites in the southeastern part of South Africa have almost no evidence of bead manufacture. For the period 290 cal BC to cal AD 310, sites with livestock are overwhelmingly also sites of bead manufacture. In subsequent periods, this pattern is not sustained.

Ostrich eggshell containers are absent from sites in eastern southernmost Africa, and from northern Botswana. They are rare in northern Namibia. They are found in greatest concentration on South Africa’s western coastal strip. Gourds (*Lagenaria sp*.) are found only within the summer rainfall area of southern Africa, which is their preferred habitat [130].

### Pottery: Was pottery exchanged? Was it made by hunter-gatherers?

We suggest that pottery was introduced into southern Africa by a small group of migrating pastoralists around 2300 years ago [131, 132, cf. 42]. Their sites stand out as having evidence of livestock, bead manufacture and mineral-tempered pottery (Map 2 Fig 6, Table 1). They may have moved into the western part of South Africa, from Namibia and Northern Botswana, through the interior Nama biome and the succulent Karoo biome and the coastal areas (cf. [15, 38]). The evidence from sites containing both ostrich eggshell containers and pottery suggests that hunter-gatherers quickly adopted pottery making (cf. [42]). It is possible that pottery was subsequently dispersed among hunter-gatherers in a combination of local innovation and exchange along existing exchange networks. This accounts for its widespread occurrence at many sites during the period 290 cal BC to cal AD 310 and for its variability in temper, decoration and shape (cf. [21]).

### Acquisition of stock and the transition to pastoralism

In the period 290 cal BC to cal AD 310, some hunter-gatherers, through their interactions with incoming pastoralists, acquired stock and became livestock-keepers (Table 2). Using ostrich eggshell containers as a proxy for hunter-gatherer presence, we suggest that most sites with livestock (60%) represent hunter-gatherers with livestock. It is upon this data that we base our reasoning that the incoming pastoralists were small in number (40% of sites represent immigrant pastoralists) (see [38] who reaches a similar conclusion based on stone tool analysis). From this point we present two scenarios, neither of which can yet be verified. Scenario 1 is that these hunter-gatherer turned livestock-keepers then gradually became the dominant pastoralist group on the landscape and are the ancestors of the historically observed Khoe pastoralist groups. Scenario 2 is that this early demic and cultural diffusion process was followed by a later migration of pastoralists and that the numbers of hunter-gatherer people with livestock remained consistently low on the landscape. Scenario 2 fits with the time line offered by geneticists for the first appearance of pastoralists with an east African origin approximately 1200–1300 years ago [133, 134].

**Table 2:** Sites that potentially represent pastoralist presence in southern Africa in the given time periods.

Site Reference Number	Site name, region and country	Cultural Affiliation as identified by excavator/past research.	Map Number
94	**Toteng 1**	Hunter-gatherers with access to trade networks with Bantu-language speaking farmers or Hunter-gatherers with livestock and Bambata pottery.	2 (290 BC–AD 310)
	North West		
	Botswana		
37	**Geduld**	Hunters on the periphery of pastoralist groups.	2 (290 BC–AD 310)
	Kunene		
	Namibia		
33	**Leopard Cave**	unknown [LSA groups]	2 (290 BC–AD 310)
	Erongo		
	Namibia		
275	**Blombos Cave**	Herder	2 290 BC–AD 310)
	southern Cape coast		
	South Africa		
28	**Snake Rock**	Hunter-gatherer stock-keepers	3 (AD 311–911)
	Erongo		
	Namibia		
32	**Miribib Shelter**	unspecified LSA groups	3 (AD 311–911)
	Erongo		
	Namibia		
100	**Lotshitshi**	LSA with pottery and cattle	3 (AD 311–911)
	North West		
	Botswana		
95	**Toteng 3**	Hunter-gatherers with access to trade networks with Bantu-language speaking farmers or Hunter-gatherers-livestock-keepers with Bambata pottery	3 (AD 311–911)
	North West		
	Botswana		
94	**Toteng 1**	Hunter-gatherers with access to trade networks with Bantu-language speaking farmers or Hunter-gatherers with livestock and Bambata pottery.	3 (AD 311–911)
	North West		
	Botswana		
235	**Blinkklipkop [BKK]**	Pastoralist [Doornfontein]	3 (AD 311–911)
	Northern Cape		
	South Africa		
285e	**KBE**	Pastoralist	3 (AD 311–911)
	Western Cape		
	South Africa		
285d	**KBDe**	Pastoralist	3 (AD 311–911)
	Western Cape		
	South Africa		
286	**Kasteelberg A [KBA]**	Pastoralist	3 (AD 311–911)
	Western Cape		
	South Africa		
287	**Kasteelberg B [KBB]**	Pastoralist	3 (AD 311–911)
	Western Cape		
	South Africa		
288	**Kasteelberg C [KBC]**	Hunter-gatherer	3 (AD 311–911)
	Western Cape		
	South Africa		
267	**Diepkloof Rock Shelter**	Hunter-gatherer mix pastoralist	3 (AD 311–911)
	Western Cape		
	South Africa		
302a	**Elands Bay Cave**	Hunters-with-sheep or pastoralist	3 (AD 311–911)
	Western Cape		
	South Africa		
294	**Die Kelders [DK1]**	hunter-gatherer-fishers	3 (AD 311–911)
	southern Cape coast		
	South Africa		
2	**Oruwanje 95/1**	unspecified LSA groups	4 (AD 912–1512)
	Kunene		
	Namibia		
37	**Geduld**	Hunters on the periphery of pastoralist groups. [Smith et al. 1995]	4 (AD 912–1512)
	Kunene		
	Namibia		
27	**Falls Rock Shelter**	Pastoralist	4 (AD 912–1512)
	Erongo		
	Namibia		
69	**Striped Giraffe Shelter**	unspecified	4 (AD 912–1512)
	Erongo		
	Namibia		
52	**!Khuiseb Delta**	Pastoralist	4 (AD 912–1512)
	Erongo		
	Namibia		
167	**Ai Tomas**	Pastoralist	4 (AD 912–1512)
	Northern Cape		
	South Africa		
94	**Toteng 1**	Hunter-gatherers with access to trade networks with Bantu-language speaking farmers or Hunter-gatherers with livestock and Bambata pottery.	4 (AD 912–1512)
	North West		
	Botswana		
95	**Toteng 3**	Hunter-gatherers with access to trade networks with Bantu-language speaking farmers or Hunter-gatherers-livestock-keepers with Bambata pottery	4 (AD 912–1512)
	North West		
	Botswana		
234	**Wonderwerk**	Pastoralist [Doornfontein]	4 (AD 912–1512)
	Northern Cape		
	South Africa		
247	**Grootrif G [GFG]**	Hunter-gatherer	4 (AD 912–1512)
	Western Cape		
	South Africa		
257	**Tortoise Cave**	unspecified LSA group	4 (AD 912–1512)
	Western Cape		
	South Africa		
286	**Kasteelberg A [KBA]**	Pastoralist	4 (AD 912–1512)
	Western Cape		
	South Africa		
287	**Kasteelberg B [KBB]**	Pastoralist	4 (AD 912–1512)
	Western Cape		
	South Africa		
291	**Heuningklip**	Pastoralist	4 (AD 912–1512)
	Western Cape		
	South Africa		
267	**Diepkloof Rock Shelter**	Hunter-gatherer	4 (AD 912–1512)
	Western Cape		
	South Africa		
316	**Nelson Bay Cave**	Herders	4 (AD 912–1512)
	Western Cape		
	South Africa		

### The distinctiveness of southeastern South Africa

**Fig 11:**
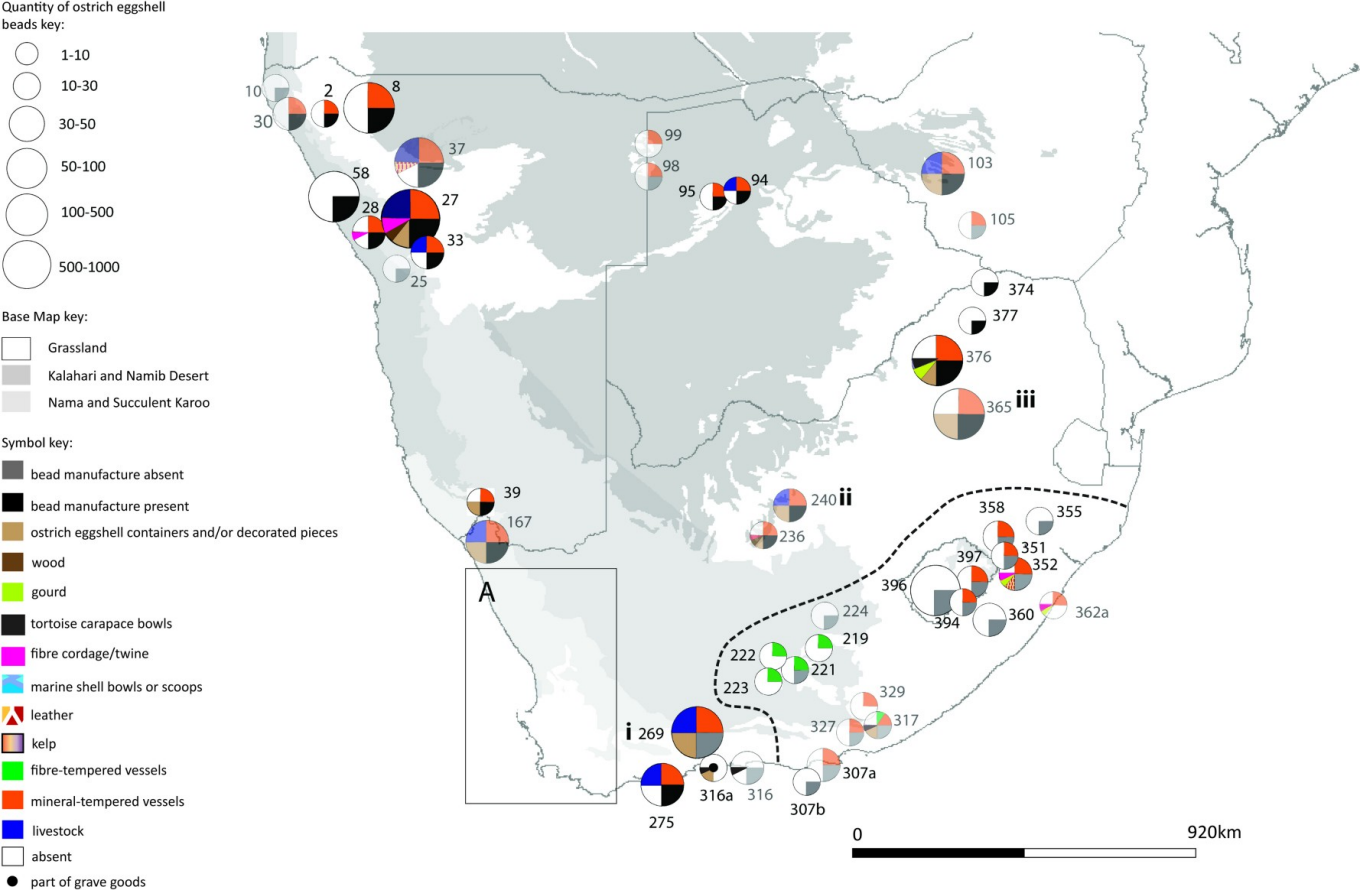
Map showing the southeastern part of South Africa and possible source areas for pottery and beads.

**Fig 12:**
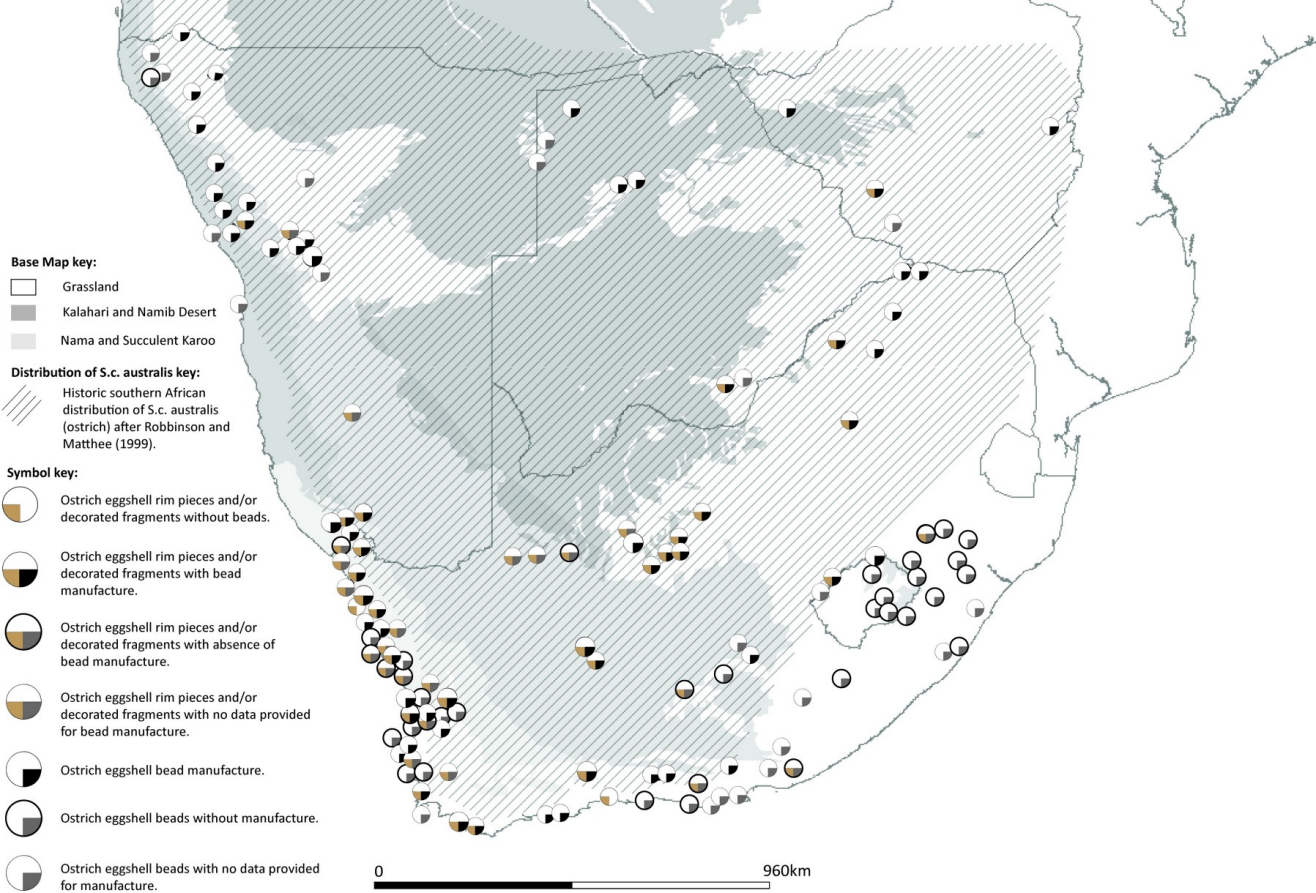
Map showing the distribution of ostrich eggshell finished beads, manufacture sites and containers against the natural habitat of the ostrich.

The southeastern part of South Africa stands out as distinct in the mapping exercise (Fig 11). In the period 290 cal BC to cal AD 310, Later Stone Age sites in the area show no evidence of ostrich eggshell bead manufacture, although almost all of the sites have evidence of ostrich eggshell beads (Fig 12). There is also no evidence of the use of ostrich eggshell containers. This area is not a natural habitat for ostriches (see [53, 55, 129] nor were these items commonly exchanged between groups (Fig 12). These sites also contain no evidence of livestock (with the exception of undated, painted rock art images of fat-tailed sheep found in low numbers) [45]. Yet they nearly all have pottery. Thin-walled mineral-tempered vessels are found in the Maloti-Drakensberg and the Thukela river valley, whilst the interior sites in the succulent Karoo biome have fibre-tempered thick-walled vessels. The mapped evidence suggests that mineral-tempered pottery was diffusing along already established social exchange networks that carried finished ostrich eggshell beads into the area. The likely source for beads and mineral-tempered pottery for sites in KwaZulu-Natal and Lesotho are area (iii) (for example, the site of Jubilee shelter, 365) approximately 400–600 km away, or area (ii) (e.g the site of Limerock 2, 240) which is a distance of approximately 400–640 km away (Fig 11). It is likely that fibre-tempered, bowl-shaped pottery in this area represents a local innovation amongst hunter-gatherers. This is the first appearance of the use of fibre temper in southern Africa. It is unlikely the pottery came from elsewhere. Mineral-tempered, wide-mouthed bowls are found at two sites in the surrounding area at approximately the same time (at sites 269 (Boomplaas) and 294 (Die Kelders) (area i, Fig 11)). The fibre-tempered bowls slightly pre-date those with mineral-temper. The area with fibre-tempered pottery might also have been more socially isolated: not all sites have finished beads and they occur in low counts of 1 to 10 beads.

### Continuing the hunter to herder debate

In recent research, Sadr [38] presented a new dataset presenting the ratios of backed stone tools to scrapers at 123 southern African archaeological sites. By comparing their ratio prior to the arrival of livestock (4000 to 2000 years ago) to that after the arrival of livestock (2000 to 1000 years ago), he concludes that a scraper-rich toolkit can be seen as a “cultural emblem” for southern Later Stone Age hunter-gatherer populations, whilst backed rich toolkits are linked to hunter-herders [38:9]. Webley [135] made a similar suggestion, that herder sites would lack formally retouched scrapers. Using this pattern, Sadr [38] recognises two small-scale migrations, one of which brought livestock and pottery along the Atlantic seaboard to the southern coast of South Africa. The other originated in the Zambezi watershed, with livestock and pottery introduced by hunter-herders but later adopted by hunter-gatherers moving in a westward direction across northern Botswana.

We have compared our data, where possible, to that of Sadr [38: Table 2]. Our results are complimentary: sites that we would classify as hunter-gatherer based on the presence of ostrich eggshell containers have scraper rich toolkits. Sadr works with a much longer time span than we do. He distinguishes two periods, before and after the arrival of livestock to the south. Further work using these two approaches in combination, for tighter and overlapping time spans, may prove productive.

Only two sites, Die Kelders (site 294) and Spoegrivier (site 165), contradict Sadr’s [38] findings. He argues that the furthest south the northern backed toolkit makers (i.e. people bringing livestock and pottery) reached was the site of Die Kelders in the southern Cape, with its backed tool rich assemblage (250 cal BC–cal AD 350, 8.9% scrapers and 58.9% backed tools) [38: Table 2]. Our interpretation is different: between 290 cal BC to cal AD 310, the site was occupied by hunter-gatherers who used ostrich eggshell containers, as well as marine and tortoise shell containers. They also used, and possibly made, mineral-tempered thin-walled pottery and manufactured ostrich eggshell beads. Later the site reflects a livestock-keeper signature, of mineral-tempered pottery and livestock. Ostrich eggshell containers are no longer used. This pattern can be interpreted in two ways: either the hunter-gatherers adopted livestock, the interpretation we favour, or an incoming group replaced them at around 300 cal AD. However, the stratigraphy at Die Kelders is disturbed [136]. It has been shown, by directly dating sheep bone, that smaller items, such as this bone, had moved from above through the Die Kelders stratigraphy into older layers [137]. It is possible that tiny backed tools, measuring 10 to 19 mm in height and a few mm in width have infiltrated the lower levels at Die Kelders. Backed tools occur in many layers at Die Kelders (layers 1 (the youngest level), 5, 7, 8, 9 and 12 (the oldest level) at the site [136: 173]) whereas scrapers (a hunter-gathers item [38]) are only found in the oldest layer, Layer 12, and are thus likely to be in situ.

At the site of Spoegriver Cave, Sadr [38: 11] notes that in Phase 1 the site had a scraper-rich stone tool assemblage (2030–1630 cal BC, 33% scrapers, 33% backed tools), and that this was replaced with a backed rich one that arrived with sheep and pottery (10–970 cal AD, 0% scrapers, 70% backed tools). Our data presents Spoegrivier as occupied by hunter-gatherers becoming livestock keepers from 290 cal BC to cal AD 310. The assemblage contains decorated ostrich eggshell containers, mineral-tempered pottery, evidence of bead manufacture and fibre/twine, and of sheep. Level 6b, the oldest level with pottery (38: 27) has one scraper (cf. [38]) and dates to this time bracket. This level has just one backed tool. In this case the large time slice used [38] may mask subtle changes through time. From approximately 300 AD, the only change to this signature is that the ostrich eggshell containers are undecorated and tortoise carapace bowls are found. We would interpret this as a case of hunter-gatherers becoming herders.

These results suggest the early presence of immigrant pastoralists at around 2300–2100 years BP. This is at odds with the earlier date of 1300 years BP for a pastoralist migration with origins in eastern Africa as suggested by geneticists–who state that this date may be an under-estimate[133](cf. [134, 138, 139]). The only second migration event we detect in the data presented here, is that of Bantu-language-speaking farmers, whose pottery appears at sites in the eastern half of southern Africa (Map 3, Fig 7 and Map 4, Fig 8). The possibility of a more recent migration (circa 1200–1300 years BP) needs further exploration, as does the possibility that the genetic clock is incorrect or an underestimate, as stated by Breton et al. [133].

Vedder’s [88] observation of Nama pastoralists with ostrich eggshell water containers warns us not to get too prescriptive about the significance of their occurrence, and is a reminder that in these interpretations we cannot rely on any one piece of evidence.

## Conclusion

Genetic and linguistic analysis shows that there was a migration of genetically distinct stock-keepers into southern Africa [131, 132, 133, 134, 138]. The challenge for us is to try to identify this in the archaeology. Based on the ethnographic, historic and archaeological record for the occurrence of ostrich eggshell water containers in southern Africa, we suggest that ostrich eggshell containers are a marker of hunter-gatherers. This appears to be confirmed in their co-occurrence at sites that are scraper rich. Through the spatial temporal mapping of the co-occurrence of ostrich eggshell containers with pottery, we reason that these two types of containers fulfilled different functions for hunter-gatherers. As hunter-gatherers became stock-keepers their use of ostrich eggshell dwindled. The data presented show that pottery spread rapidly to sites across southern Africa from 2300 years ago. We suggest that pottery, first introduced by a small group of immigrant pastoralists, was quickly adopted and made by hunter-gatherers and then spread by cultural diffusion (cf. [30]). Through the mapping of bead manufacture sites relative to sites with evidence of finished beads only, we are able to argue that both mineral-tempered pottery and beads may have been brought to the southeastern part of South Africa on pre-existing hunter-gatherer exchange networks (cf. [125]). By the same reasoning, it appears that the manufacture of fibre-tempered vessels was unique to hunter-gatherers during this period. We raise the hypothesis that sites with livestock, pottery and ostrich eggshell containers represent autochthonous hunter-gatherers becoming livestock-keepers. It is possible that this group then became the dominant pastoralists on the landscape, eventually giving rise to the Khoe-pastoralists as known from the historic period.

## Supporting information

S1 Map KMZ file to show the temporal and geographic spread of the data contained within Database 1. (KML)

S1 Table The sources used in the compilation of Database 1. (DOCX)

S2 Table The material from six of the sites in Database 1, recorded during museum collection visits, 2017. (DOCX)

S3 Table Historic and ethnographic accounts of the use of containers in southern Africa, 16^th^ century AD to the present. (DOCX)

S1 Appendix Database 1 and 2. (XLSX)

S2 Appendix Spoegriver (165) and Jakkalsberg B (170). Examples of fibre-tempered and mineral tempered pottery, twine and ostrich eggshell photographed during museum collection visits, 2017.
(TIF)

S3 Appendix Blinkklipkop (235) and dikbosch 1 (236). Examples of mineral tempered pottery and ostrich eggshell photographed during museum collection visits, 2017.
(TIF)

S4 Appendix Haaskraal (221) and Die Kelders (294). Examples of fibre-tempered and mineral tempered pottery, twine and ostrich eggshell photographed during museum collection visits, 2017.
(TIF)
