# Supplementary material for: A southern African archaeological database of organic containers and materials, 800 cal BC to cal AD 1500: Possible implications for the transition from foraging to livestock-keeping
Source: PLoS One. 2020 Jul 8;15(7):e0235226. doi: 10.1371/journal.pone.0235226 (PMC7343145; doi:10.1371/journal.pone.0235226)
Supplement: S1 Table — (DOCX) [file pone.0235226.s002.docx]

| Publication Source | Number of published reports |
| --- | --- |
| The South African Archaeological Bulletin | 52 |
| Southern African Humanities (earlier name of journal: Annals of the Natal Museum) | 27 |
| South African Field Archaeology | 10 |
| Cimbebasia | 7 |
| African Archaeological Review | 7 |
| Journal of Archaeological Science | 6 |
| Radiocarbon | 5 |
| Before Farming | 4 |
| Journal of African Archaeology | 4 |
| Botswana Notes and Records | 4 |
| South African Journal of Science | 3 |
| Researches of the National Museum, Bloemfontein. | 3 |
| Journal of Island and Coastal Archaeology | 2 |
| Journal of Arid Environments | 2 |
| South African Archaeological Society: Goodwin Series | 2 |
| Annals of the South African Museum | 2 |
| Antiquity | 2 |
| Archaeometry | 1 |
| PaleoAnthropology | 1 |
| PlosOne | 1 |
| Nyame Akuma | 1 |
| Annals of the Eastern Cape Museum | 1 |
| Annals of the Ditsong National Museum of Natural History | 1 |
| Journal of Anthropological Archaeology | 1 |
| World Archaeology | 1 |
| Azania: Archaeological Research in Africa | 1 |
| Pula: Botswana Journal of African Studies | 1 |
| Current Anthropology (Curr Anthropol.) | 1 |
| Madoqua | 1 |
| Science | 1 |
| South African History Journal | 1 |
| Zimbabwea | 1 |
| Books | 6 |
| Edited Books | 5 |
| Unpublished University dissertations or theses | 13 |
| Monographs | 16 |
| Unpublished Government Reports | 1 |

S1 Table: List of key sources used to compile Database 1.
