# Supplementary material for: A southern African archaeological database of organic containers and materials, 800 cal BC to cal AD 1500: Possible implications for the transition from foraging to livestock-keeping
Source: PLoS One. 2020 Jul 8;15(7):e0235226. doi: 10.1371/journal.pone.0235226 (PMC7343145; doi:10.1371/journal.pone.0235226)
Supplement: S2 Table — (DOCX) [file pone.0235226.s003.docx]

| ***Site reference Number (Database 1)*** | ***Site and region*** | ***uncal. radiocarbon date*** | ***Items selected for further documentation*** | ***National Repository and Museum Site Accession**** |
| --- | --- | --- | --- | --- |
| **165** | **Spoegrivier**  Namaqualand, Northern Cape, South Africa. | 1450±50 Pta-6750  1250±50 GrA-9027  1490±50 GrA-9030  1890±50 GrA-9029  1900±50 GrA-9032  1900±50 GrA-9028  1930±50 Pta-6749  2105±65 | - Ostrich eggshell containers - Twine - Mineral-tempered pottery. | McGregor Museum, Kimberley, Northern Cape, South Africa.  Museum Site Accession:  Spoeg River (LE. W). |
| **169, 170** | **Jakkalsberg A and B**  Richtersveld, Northern Cape, South Africa. | 1420±25 Pta-6122  1380±50 Pta-6101  1330±60 Pta-5958  1300±25 Pta-6100 | - Lugged mineral-tempered pottery. | McGregor Museum, Kimberley, Northern Cape, South Africa.  Museum Site Accession:  Jakkalsberg A & B (Cultural). |
| **235** | **Blinkklipkop**  Kuruman Hills, Northern Cape, South Africa. | 1160±50 Pta-2840  1150±40 Pta-2835 | - Ostrich eggshell containers. - Mineral-tempered pottery. | McGregor Museum, Kimberley, Northern Cape, South Africa.  Museum Site Accession:  BKK 6549. |
| **236** | **Dikbosch 1 & 2**  Ghaap Escarpment, Northern Cape, South Africa. | 1720±40 Pta-3413  1570±40 Pta-3412 | - Ostrich eggshell containers. - Mineral-tempered pottery. | McGregor Museum, Kimberley, Northern Cape, South Africa.  Museum Site Accession:  DIK 6480. |
| **221** | **Haaskraal (HAA)**  Karoo, Northern Cape, South Africa. | 1570±50 GrA-13542  1400±50 GrA-13540  1180±70 SMU-1789  1140±60 SMU-1790  545±45 SMU-1636  544±43 SMU-1636 | - Ostrich eggshell containers. - Lugged mineral-tempered pottery and grass-tempered pottery. | Iziko South African Museum, Cape Town, South Africa.  Museum Site Accession:  SAM-AA-9099. |
| **294** | **Die Kelders (DK1)**  southern Cape, South Africa. | 1960±85 GX-1688  1960±85 GX-1687  1325±60 OxA-3860  1290±60 OxA-3961 | - Ostrich eggshell containers. - Marine Shell bowl with ochre. - Mineral-tempered pottery. | Iziko South African Museum, Cape Town, South Africa.  Museum Site Accession:  SAM-AA-8725. |

S2 Table: A list of six sites, their organic and pottery assemblages and associated dates that were recorded at South African Museums.

* No permits were required for the described study, which complied with all relevant regulations.
