# Supplementary material for: A southern African archaeological database of organic containers and materials, 800 cal BC to cal AD 1500: Possible implications for the transition from foraging to livestock-keeping
Source: PLoS One. 2020 Jul 8;15(7):e0235226. doi: 10.1371/journal.pone.0235226 (PMC7343145; doi:10.1371/journal.pone.0235226)
Supplement: S3 Table — (DOCX) [file pone.0235226.s004.docx]

S3 Table: Historic and ethnographic accounts of the use of containers in southern Africa, 16^th^ century AD to the present.

| Southern African Cultural Group | Number of accounts captured | Dates captured |
| --- | --- | --- |
| Nama | 9 | 1806-1956 |
| Damara | 1 | 1834 |
| G\|ana and G\|\|wi | 2 | 1959-60s, 1993 |
| Nharo | 2 | 1930, 1968-83 |
| Auen | 1 | 1930 |
| Ju’\|’hoansi (!Kung) | 3 | 1914-19, 1930-61 |
| ǂAuin | 3 | 1908-1911 |
| Kxoé | 1 | 1911-1990s |
| Cape Khoe* | 11 | 1617-1713, 1770-79 |
| \|Xam* southern San | 8 | 1704-1812, 1938 |
| Koranna* | 3 | 1801 – 1820 |
| Herero | 5 | 1884-87, 1967, 1974 |
| Himba | 3 | 1967-86, 1991 |
| Gonaqua* | 4 | 1772-82, 1829 |
| !Xhosa | 12 | 1593, 1777-1855, 1932, 1974 |
| Mpondo | 5 | 1782, 1899, 1949-74 |
| Bomvana | 2 | 1931, 1974 |
| Baca | 1 | 1974 |
| Thembu | 4 | 1825-34, 1974 |
| Hlubi | 1 | 1974 |
| Amazizi | 1 | 1824 |
| Xesibe | 1 | 1974 |
| Zulu | 3 | 1900, 1981, 2018 |
| Swazi | 3 | 1930, 1959-81 |
| **24** | **89** | **1593 - 2018** |
