# Supplementary figures and images for: A southern African archaeological database of organic containers and materials, 800 cal BC to cal AD 1500: Possible implications for the transition from foraging to livestock-keeping
Source: PLoS One. 2020 Jul 8;15(7):e0235226. doi: 10.1371/journal.pone.0235226 (PMC7343145; doi:10.1371/journal.pone.0235226)

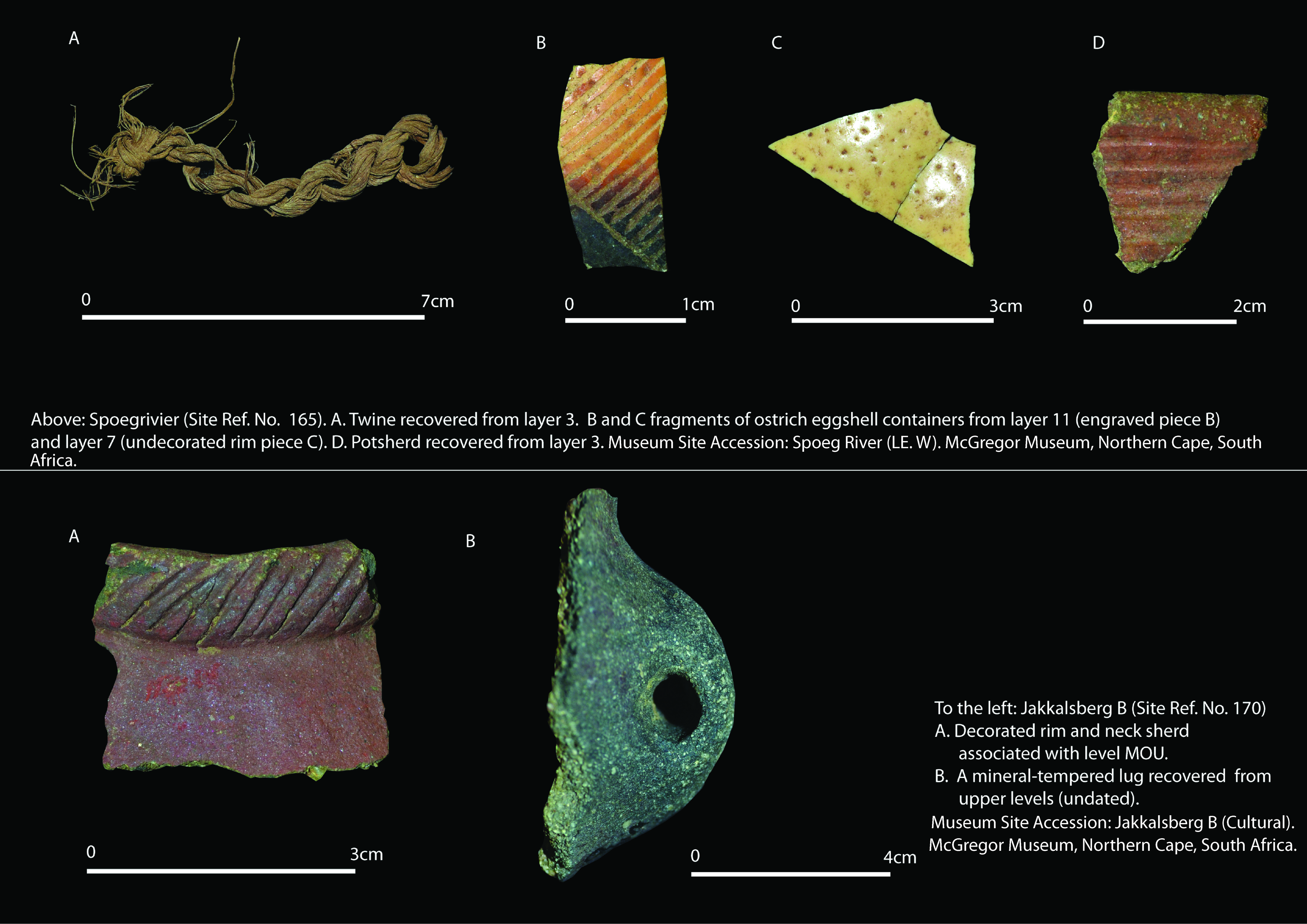

Supplement: S2 Appendix — Examples of fibre-tempered and mineral tempered pottery, twine and ostrich eggshell photographed during museum collection visits, 2017. (TIF) [file pone.0235226.s006.tif]

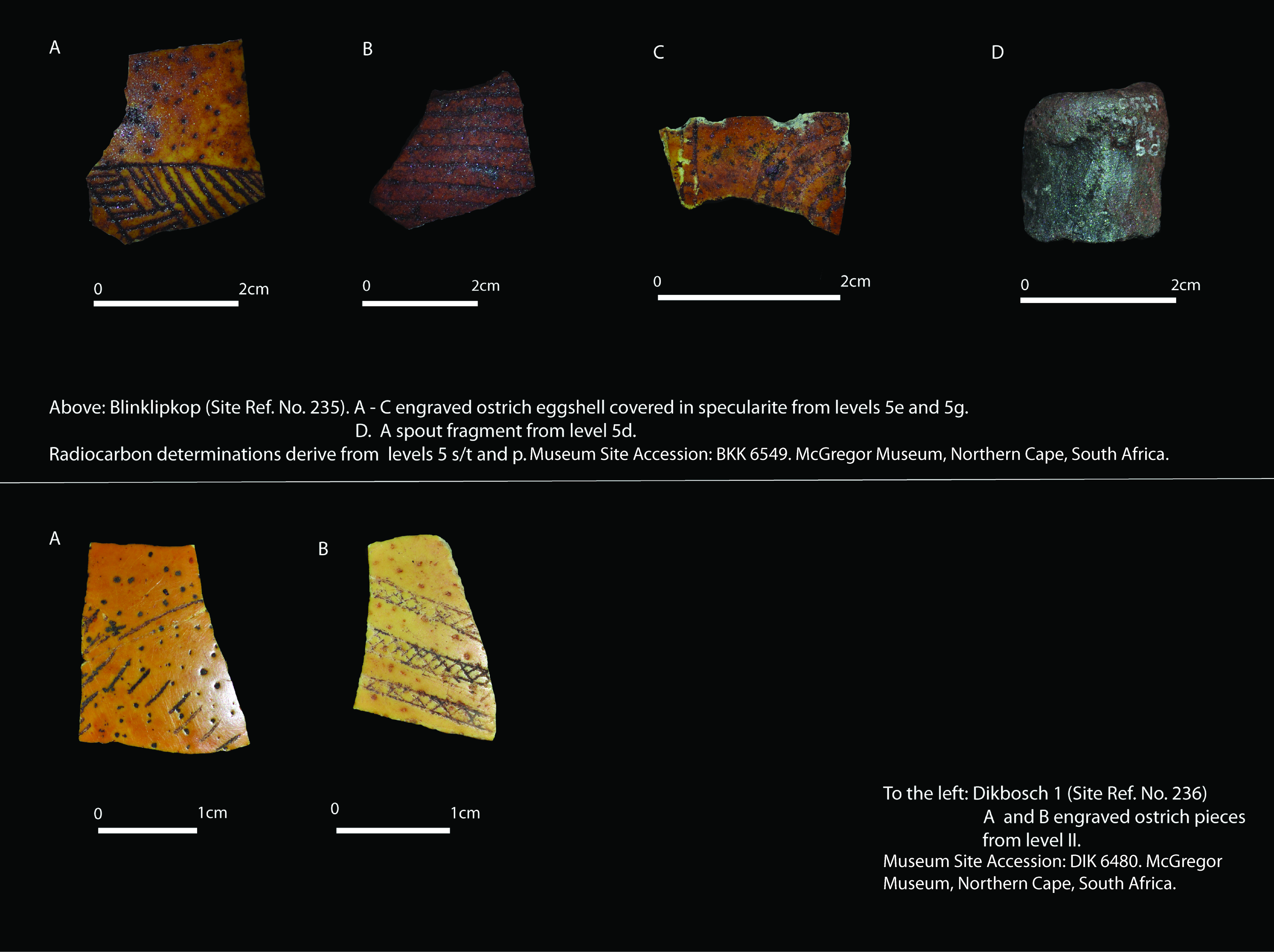

Supplement: S3 Appendix — Examples of mineral tempered pottery and ostrich eggshell photographed during museum collection visits, 2017. (TIF) [file pone.0235226.s007.tif]

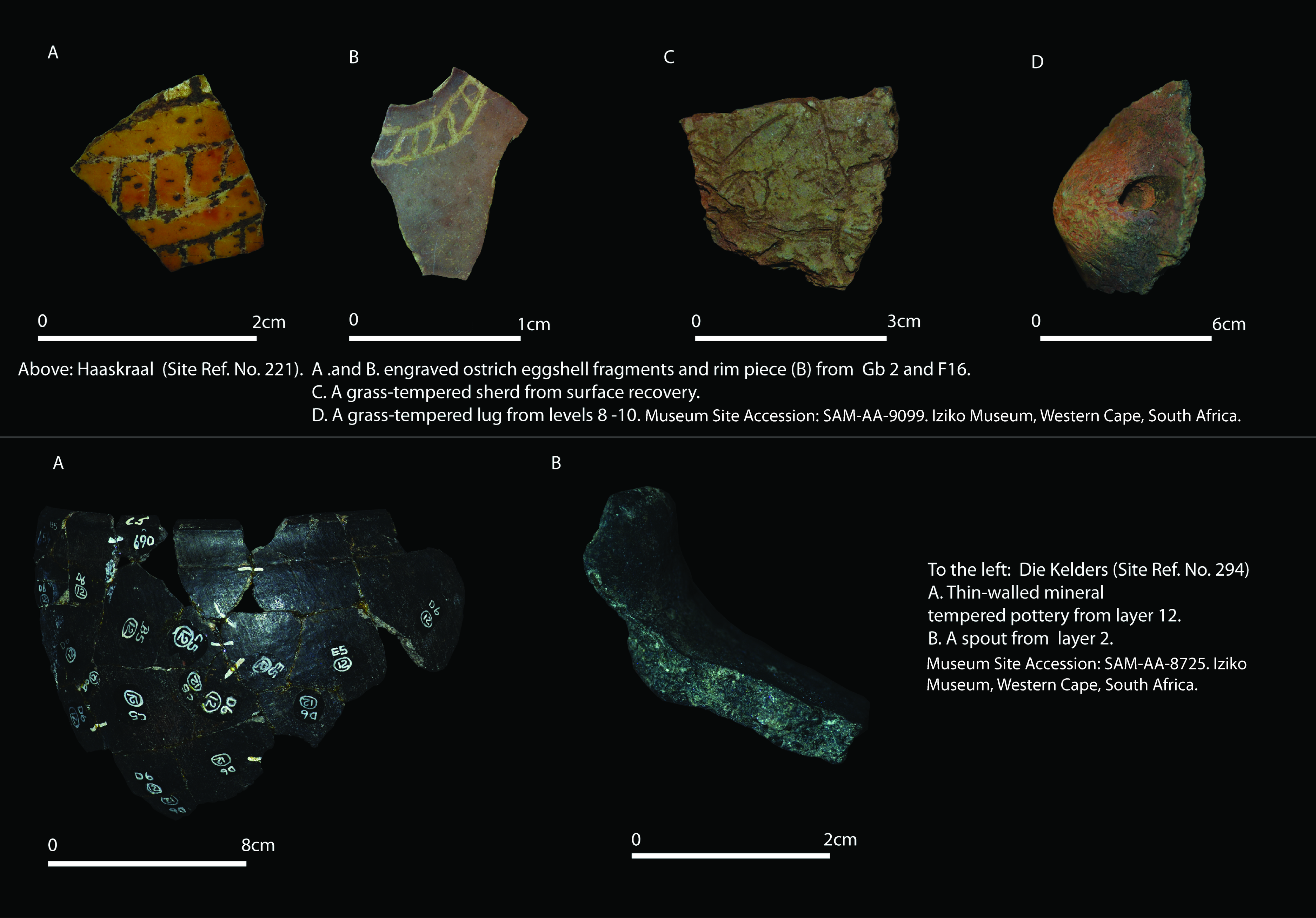

Supplement: S4 Appendix — Examples of fibre-tempered and mineral tempered pottery, twine and ostrich eggshell photographed during museum collection visits, 2017. (TIF) [file pone.0235226.s008.tif]
